# Simple and Efficient Synthesis of *N*-Succinimidyl-4-[^18^F]fluorobenzoate ([^18^F]SFB)—An Important Intermediate for the Introduction of Fluorine-18 into Complex Bioactive Compounds

**DOI:** 10.3390/ph17121723

**Published:** 2024-12-20

**Authors:** Viktoriya V. Orlovskaya, Olga S. Fedorova, Nikolai B. Viktorov, Raisa N. Krasikova

**Affiliations:** 1 N. P. Bechtereva Institute of the Human Brain, Russian Academy of Sciences, 197022 St. Petersburg, Russia; orlovskaya@ihb.spb.ru (V.V.O.);; 2 Faculty of Chemical and Biotechnology, St. Petersburg State Institute of Technology (Technical University), 190013 St. Petersburg, Russia; kolki@mail.ru

**Keywords:** fluorine-18, copper-mediated radiofluorination, [^18^F]SFB, prosthetic group, one-step synthesis, cartridge-based purification

## Abstract

**Background:** *N*-succinimidyl-[^18^F]fluorobenzoate ([^18^F]SFB) is commonly prepared through a three-step procedure starting from [^18^F]fluoride ion. A number of methods for the single-step radiosynthesis of [^18^F]SFB have been introduced recently, including the radiofluorination of diaryliodonium salts and the Cu-mediated ^18^F-fluorination of pinacol aryl boronates and aryl tributyl stannanes, but they still have the drawbacks of lengthy product purification procedures. In the present work, two approaches for the direct labeling of [^18^F]SFB from diaryliodonium (DAI) salt (**4**) and pinacol aryl boronate (**6**) are evaluated, with a major focus on developing a fast and simple SPE-based purification procedure. **Methods:** DAI salt precursor **6** was labeled employing the common “minimalist” approach with a two-step reaction heating sequence. The Cu-mediated radiofluorination of **4** was accomplished using Bu_4_NOTf as a phase transfer catalyst for the elution of [^18^F]fluoride, followed by radiofluorination in the same solvent. Several types of SPE cartridges were tested in the elution and SPE procedures. **Results:** The Cu-mediated ^18^F-fluorination of the pinacol aryl boronate precursor afforded a higher RCC of 56 ± 3% (n = 7), making it better suited for the one-pot synthesis of [^18^F]SFB. SPE-based purification was achieved using cation exchange and reverse-phase polymer resin cartridges, connected in series. In a full-batch test, [^18^F]SFB was obtained with an RCY of 30% (n. d. c.), RCP > 99%, A_m_ 96–155 GBq/µmol, and a synthesis time of ≤35 min. **Conclusions:** Compared to other published methods, [^18^F]SFB production via the Cu-mediated radiofluorination of pinacol aryl boronate precursor provides significant time and cost savings, coupled with an ease of implementation.

## 1. Introduction

The development of target-specific PET radiotracers is the ultimate end-point of PET methodology research, and a lot of effort is directed towards this goal at any given time; however, it is not an easy feat to achieve, given the difficulty of the task itself and the chemical complexity of potential tracers. For the targeted molecular imaging of cancer, there has been considerable interest in peptide-based radiotracers; the majority of them are labeled with generator-produced ^68^Ga (T_1/2_ 68 min, 89% β^+^) or other radiometals [1]. However, over the last two decades, an increasing number of [^18^F]-labeled peptides have been suggested as attractive alternatives to radiometal-labeled peptides [2,3]. Among the positron-emitting radionuclides in routine use, ^l8^F (97% β^+^) is particularly attractive as a labeling entity due to its relatively long half-life (109.7 min) enabling the lengthy multi-step syntheses often required for labeling complex biomolecules. Another advantage is that fluorine-18 is a low-energy positron emitter (0.635 MeV) and a corresponding shorter positron range (2.4 mm) enables higher theoretical spatial image resolution. The radionuclide is readily available in sufficient quantities (up to 900 GBq) through ^18^O(p,n)^18^F nuclear reaction in high-pressure ^18^O-enriched water targets of commercial cyclotrons. Using no-carrier-added [^18^F]fluoride, the corresponding radiotracers can be prepared with the high molar activity (1–10 Ci/µmol) required for the imaging of the receptors over-expressed in the membranes of tumor cells. Out of several approaches for the direct incorporation of fluorine-18 into complex biomolecules [3], labeling via [^18^F]AlF chelation has been found to be efficient in the preparation of a variety of peptides and proteins (for a recent review, see [4]). This simple and versatile procedure is based on the formation of an [^18^F]AlF complex via the direct reaction of an aqueous [^18^F]F^−^ with AlCl_3_ in an acidic milieu (pH 4), followed by the complexation with NOTA or another suitable chelator conjugated to a molecule of interest. However, the chelation reaction requires elevated reaction temperatures (100–120 °C), imposing limitations on the ^18^F labeling of heat-sensitive biomolecules. Also, special care must be taken in case of acid-sensitive molecules.

So far, in the majority of cases, peptide labeling has been achieved by using ^18^F-containing prosthetic groups—small, bifunctional molecules that are first labeled with ^18^F with subsequent attachment to the active site of the peptide via a conjugation reaction. Among a plethora of the different prosthetic groups for such bioconjugation [5], the most common one is *N*-succinimidyl-4-[^18^F]fluorobenzoate ([^18^F]SFB). For example, this reagent is known to be effective in the radiolabeling of annexin V, cyclic RGD peptides, neurotensin, single-domain antibodies [6,7,8,9], and many other biomolecules.

**Scheme 1:**
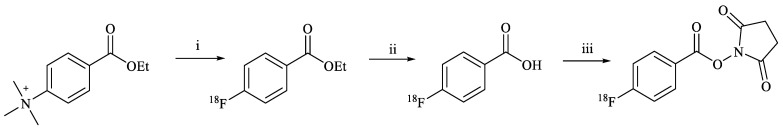
Three-step radiosynthesis of [^18^F]SFB: (i) K^+^/K_2.2.2_./^18^F^−^, CH_3_CN, 90 °C; (ii) TPAH, 120 °C; (iii) TSTU, 90 °C [10].

The synthesis of [^18^F]SFB was first reported in 1992 by Vaidyanathan and Zalutsky [10]. The radiosynthesis was based on a three-step procedure starting from the incorporation of [^18^F]fluoride into 4-formyl-*N*,*N*,*N*-trimethylanilinium-triflate, followed by oxidation to form 4-[^18^F]fluorobenzoic acid and subsequent dicyclohexylcarbodiimide (DCC) activation to produce [^18^F]SFB. The total synthesis time, including HPLC purification, was 100 min, and the decay-corrected radiochemical yield (RCY) was 25%. In the following publication by Wester et al. [11], the synthesis was modified, as follows: ethyl-4-(trimethylammonium)benzoate was used instead of the benzaldehyde precursor, and *O*-(*N*-succinimidyl-*N*,*N*,*N*′,*N*′-tetramethyluronium tetrafluoroborate (TSTU) was applied to convert the benzoic acid intermediate to [^18^F]SFB (Scheme 1).

Currently, this procedure is used in the majority of cases when [^18^F]SFB is produced using modern automated synthesis modules. In order to increase the quantity of the radiotracer recovered at the end of the synthesis (EOS), significant efforts have been undertaken to shorten the synthesis time and simplify the purification by replacing the HPLC procedure with solid-phase extraction (SPE) using disposable cartridges. An excellent summary of the reported automated radiosyntheses of [^18^F]SFB using ethyl 4-(trimethylammonium)benzoate triflate (one- or two-pot procedures) has been provided in a recent paper by Al-Qahtani et al. [12]. To give one example, a one-pot synthesis procedure using the AllinOne cassette-based automation platform provided a decay-corrected RCY of 44 ± 4% (n = 13), with an average synthesis time of 54 min [9]. Similarly, the “two-pot” synthesis procedure realized on the GE FASTLab automated module took 57 min and yielded [^18^F]SFB in about 42% decay-corrected RCY (n = 24) [6].

Despite the progress made, the most efficient strategy for radiolabeling SFB with fluorine-18 would be a late-stage approach consisting of incorporating fluorine-18 in the final step of the synthesis. A one-step synthesis of the [^18^F]SFB via fluorodenitration of *N*-succinimidyl-4-nitrobenzoate using potassium fluoride and Kryptofix as a phase-transfer catalyst (PTC) was attempted already in 1992 [10]. However, while most of the nitro precursor was consumed, none of the desired product was produced. The recent development of the comparatively simple, efficient, and innovative late-stage radiofluorination approaches [13,14] opens up new possibilities for the direct introduction of the ^18^F-label into the aromatic compounds with non-activated aryl scaffolds. Those evaluated for the single-step radiosynthesis of the [^18^F]SFB included radiofluorination of diaryliodonium salts [15,16] or iodonium ylides [17] as precursors, as well as the copper-mediated ^18^F-fluorination of pinacol esters of arylboronic acids (ArylBPin) [18,19] or aryl tributyl stannanes [20]. The following modest to good decay-corrected RCYs have been achieved for copper-mediated approaches: 22–34% for ArylBPin precursor [19] and 42 ± 4% for the stannylated one [20]. However, both methods used semi-preparative HPLC purification for the [^18^F]SFB, resulting in a rather long synthesis of 68 to 83 min [19], and thus negating the advantages of the one-step approach. The implementation of SPE purification for this particular procedure instead of HPLC could facilitate the automation and subsequent expansion of the use of this prosthetic group in the radiolabeling of peptides and other biomolecules.

In this paper, we focus our efforts towards development of an optimized one-step radiosynthesis of the [^18^F]SFB starting from diaryliodonium (DAI) salt and the boronic acid pinacol ester (ArylBPin) as labeling precursors with the isolation of the target product using disposable SPE cartridges. The potential of the SPE-purified [^18^F]SFB in peptide labeling is evaluated in a small-scale conjugation reaction with the readily available tripeptide, glutathione, as a model for future coupling reactions.

## 2. Results and Discussion

### 2.1. Synthesis of [^18^F]SFB via Diaryliodonium Salt Precursor (Method A)

Of the several late-stage radiofluorination approaches described for the incorporation of [^18^F]fluoride into non-activated aromatic arenes [13,14], we first evaluated the applicability of diaryliodonium (DAI) salts as labeling precursors for [^18^F]SFB. The use of this class of precursors, pioneered by the Pike group [21], proved to be an effective route for the preparation of a number of PET radiotracers (for review see [22]). Due to their electron-deficient nature at the iodine atom and the simultaneous presence of an excellent leaving group in the form of a Ph-I fragment, the diaryliodonium salts exhibit exceptional reactivity in the nucleophilic fluorination reaction without the need for further activating groups. The radiofluorination of DIA salts containing phenyl-, 4-tolyl-, 2-thienyl-, 4-anisyl-, and 2,4,6-trimethoxyphenyl- proceeds via a thermal decomposition step without the need for any catalysts. These so-called “spectator” aryl groups play an important role in directing the [^18^F]fluoride ion to the electron-rich aryl leaving group. The applicability of this approach to [^18^F]SFB synthesis was first demonstrated in 2008 by Carrol et al. The results were presented as a short conference abstract [15] without detailed information. Using (4-((2,5-dioxopyrrolidin-1-yloxy)carbonyl)phenyl)(thiophen-2-yl)iodonium trifluoroacetate with thiophenol as a “spectator” group, the crude RCY of [^18^F]SFB was 13-23% (n = 4) [15]. The target product was not isolated and no further reports on the subject have been published. More recently, the synthesis of [^18^F]SFB was realized using aryl(2,4,6-trimethoxyphenyl)iodonium tosylate as the radiolabeling precursor [16] under Kryptofix-mediated fluorination (K_2.2.2._/K_2_CO_3_, DMA, 140 °C, 10 min). RCY was evaluated by HPLC analysis of the crude reaction mixture (60% CH_3_CN/H_2_O with 0.01% Et_3_N (*v*/*v*)), and was comparable to the previously published results—20 ± 3% (n = 3).

**Scheme 2:**
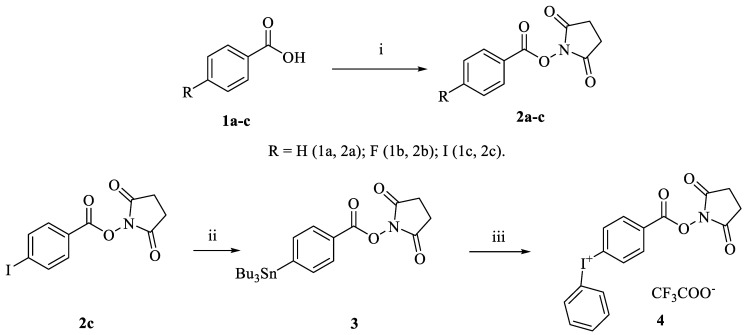
Synthesis of [^19^F]SFB (**2b**) and (4-((2,5-dioxopyrrolidin-1-yloxy)carbonyl)phenyl)(phenyl)iodonium trifluoroacetate (**4**). Reagents and conditions: (i): di(*N*-succinimidyl) carbonate, Et_3_N, CH_3_CN, 20 °C, 20 h; yield **2a**—41%, **2b**—44%, **2c**—73%; (ii): *n*-Bu_6_Sn_2_, Pd(PPh_3_)_4_, toluene-DMF, reflux, 4 h, 62%; (iii): PhI(OC(O)CF_3_)_2_, DCM, 20 °C, 40 h, 28%.

To elaborate on this result, we have selected the DIA salt precursor, (4-((2,5-dioxopyrrolidin-1-yloxy)carbonyl)phenyl)(phenyl)iodonium trifluoroacetate (**4**), which was prepared by a three-step synthesis (Scheme 2). This synthetic procedure also allowed the isolation of the required reference standard [^19^F]SFB (**2b**). The detailed synthesis is described in the Materials and Methods section.

**Scheme 3:**
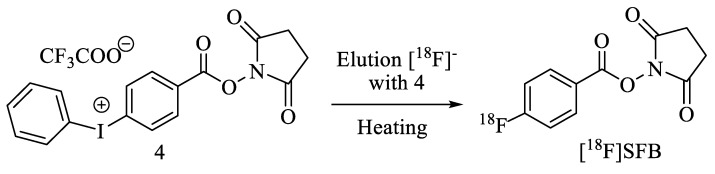
Labeling of [^18^F]SFB via diaryliodonium salt precursor **4** (Table 1).

In order to find optimal conditions for the radiofluorination of **4** (Scheme 3), several combinations of reaction solvents and heating conditions were investigated (Table 1). The synthesis started with the trapping/elution of the [^18^F]fluoride on an anion-exchange cartridge to convert it into the reactive species more useful for the nucleophilic substitution. Based on our previous experience with DAI salt precursors ((piperonyl)(phenyl)iodonium *p*-toluenesulfonate and bromide) [23]), we applied the so called “minimalist” protocol proposed by Richarz et al. [24] for the elution step. The solution of DAI salt in organic solvent (most commonly MeOH) can be used to directly elute [^18^F]fluoride retained on the anion exchange resin; after the rapid removal of MeOH, the radiofluorination is performed in a suitable aprotic solvent without the need for the phase transfer catalyst or additional base. The practical applicability of this fast and exceptionally convenient procedure has been previously confirmed [22,23]. However, when using this approach in combination with traditionally used “basic” QMA or PS-HCO_3_ anion exchange cartridges, we observed the decomposition of **4** (Figure S26B), as this compound appears to be very sensitive to basic conditions. To avoid this, we chose to use the weak anion-exchange cartridge Oasis WAX 1 cc (30 mg). In this case, the elution of the trapped [^18^F]fluoride with the methanolic solution of **4** (18 µmol) proceeded without any decomposition of the precursor, as confirmed by HPLC analysis (Figure S26C). However, after overcoming this first hurdle, the removal of MeOH presented the next problem since the precursor **4** also decomposed during evaporation of MeOH preceding the radiofluorination step, and evaporation to dryness resulted in the complete decomposition of **4**. To solve this problem, we applied the following two-step heating procedure: the reaction mixture was heated at 75 °C for 10 min under nitrogen flow to remove most of the MeOH, followed by a second round of heating at 120 °C for 15 min with the reactor vessel sealed. Most of the MeOH was removed during the first heating step, while the radiofluorination step was performed at 120 °C for 15 min in the mixture of the protic (MeOH) and aprotic solvent (see Table 1 for different solvents). The best solvent combination was MeOH (0.2 mL)/CH_3_CN (0.5 mL) (Entry 4, Table 1), giving [^18^F]SFB in up to 36% RCC (radioTLC). However, as in the other reports [15,22], a high variability in the radiofluorination yields was observed between the individual runs. This variability, coupled with the expected dependence of the yields on the salt anion [22,23], makes this approach less amenable to the development of a one-pot synthesis procedure of the [^18^F]SFB.

### 2.2. Synthesis of [^18^F]SFB via Cu-Mediated Radiofluorination of ArylBPin Precursor ***6*** (Method B)

Another strategy allowing the one-step radiosynthesis of [^18^F]SFB is the copper-mediated radiofluorination, which was originally introduced by the Gouverneur group for pinacol aryl boronates (ArylBPin) precursors [25]. In the last decade, the Cu-mediated approach using a commercially available Cu(OTf)_2_(Py)_4_ catalyst has been successfully applied to the preparation of a range of radiotracers containing non-activated aryl (and heteroaryl) scaffolds and shown to be easily implemented in a variety of the automated synthesis modules (for recent reviews, see [14,26]). The feasibility of this approach for the synthesis of [^18^F]SFB was investigated using aliquot labeling conditions [18]. An aliquot of 20–30 MBq of azeotropically dried [^18^F]KF/K_2.2.2._ complex in 10–50 μL of acetonitrile was allowed to react with the corresponding ArylBpin precursor (2,5-dioxopyrrolidin-1-yl-4-(4,4,5,5-tetramethyl-1,3,2-dioxaborolan-2-yl)benzoate) in the presence of a copper catalyst. The radiofluorination was performed in DMA (300 μL) at 110 °C for 20 min under air atmosphere and resulted in an RCC of 61 ± 13% (n = 4) as determined by radio-TLC and radio-HPLC. However, the RCC values determined from aliquot labeling tests are often overestimated [27] because they do not take into account losses of [^18^F]fluoride on the inner walls of the reaction vessel during the azeotropic drying step. In addition, aliquot labeling procedures use lower amounts of base (e.g. K_2.2.2._/K_2_CO_3_) relative to other reactant concentrations, which is critical for reactions such as the base-sensitive Cu-mediated radiofluorination [28]. As a result, considerable effort is required to extrapolate results from aliquot labeling syntheses to a full batch automated radiotracer preparation. Recently, the radiolabeling of the [^18^F]SFB using the same ArylBPin precursor [18] has been translated to an automated radiosynthesizer TRACERLab FX2N [19]. Reactive [^18^F]fluoride was obtained using KOTf/K_2_CO_3_ as the PTC; Cu-mediated labeling at 90 °C for 15 min in DMI produced [^18^F]SFB with 13–20% (n = 3) isolated RCY with the synthesis time in the range of 68–83 min, including HPLC purification. Unfortunately, the reported procedure involved the following very small amounts of radioactivity: 2–10 MBq in the manual synthesis using a microliter scale radiofluorination in HPLC vials (RCC 43% by radio-TLC) and 10–20 MBq in the automated production.

In addition to the ArylBPin precursors, the copper-mediated radiofluorination of a stannyl derivative, *N*-succinimidyl 4-(tri-*n*-butylstannyl)benzoate, was investigated as a route to [^18^F]SFB [20]. Using Et_4_NOTf as PTC, the radiofluorination was performed at 140 °C for 10 min in DMA, followed by product isolation via cartridge-based purification. The automated procedure on the NEPTIS kit-based synthesizer yielded metal-free [^18^F]SFB with a decay-corrected RCY of 44 ± 4%. However, an additional HPLC purification was required to remove the unlabeled UV impurity, *N*-benzoyloxy succinimide, formed in the protodestannylation process that often accompanies Cu-mediated radiofluorinations [29]. The presence of this impurity has been shown to have a negative effect on the coupling reaction of [^18^F]SFB with nanobodies [20].

Based on the methods summarized in Table 2 from the literature sources discussed above, and our own experience with the copper-catalyzed radiofluorinations of ArylBPin precursors [23,30,31,32], we focused our efforts on optimizing the radiolabeling conditions of [^18^F]SFB and replacing the tedious HPLC purification with a cartridge-based procedure that is much more attractive for the automation.

**Scheme 4:**
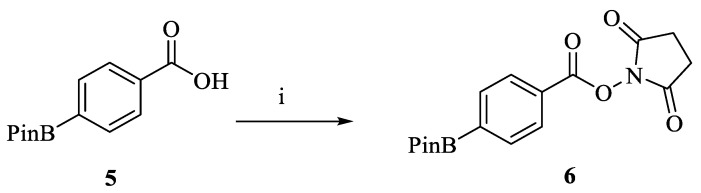
Synthesis of 2,5-dioxopyrrolidin-1-yl 4-(4,4,5,5-tetramethyl-1,3,2-dioxaborolan-2-yl)benzoate (**6**). Reagents and conditions: (i): di(*N*-succinimidyl) carbonate, Et_3_N, CH_3_CN, 20 °C, 20 h, 51%.

The precursor for radiolabeling, 2,5-dioxopyrrolidin-1-yl 4-(4,4,5,5-tetramethyl-1,3,2-dioxaborolan-2-yl)benzoate **6** (Scheme 4), was prepared in one step from commercially available materials in 51% yield (Scheme 4). The detailed synthesis is described in Section 3.

**Scheme 5:**
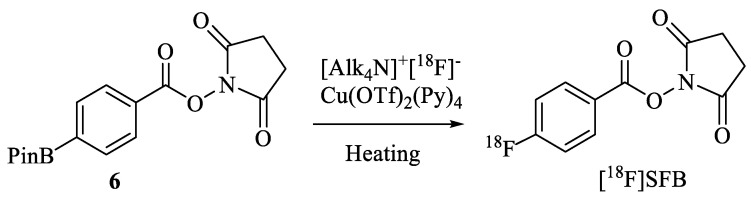
Labeling of the [^18^F]SFB using arylboronic acid pinacol ester precursor **6**; [Alk_4_N]^+^[^18^F]^−^-[Et_4_N]^+^[^18^F]^−^ or [Bu_4_N]^+^[^18^F]^−^ (Table 3).

Despite the proven efficacy of the Cu-mediated approach as demonstrated in small-scale radiolabeling [25], its implementation for full-batch tracer production exposed the problem of the sensitivity of the process to basic conditions encountered when using the classic [^18^F]KF/K_2.2.2._ fluorination with K_2_CO_3_ as the base, as demonstrated by Zlatopolskiy et al. [28]. To address this issue, several novel protocols have been developed for the reprocessing of [^18^F]fluoride by introducing less basic or neutral PTC agents in organic solvents, which not only allow the radiofluorination efficiency to be improved but also the elimination of the traditional azeotropic drying step [33,34]. Furthermore, the use of a solution of the Et_4_NHCO_3_ in alcohol, as proposed in [34], allowed a high recovery rate of the radionuclide from the anion exchange cartridge (eluted in the direction opposite to the loading) and a high efficiency for the Cu-mediated fluorination of ArylBPin precursors when the same alcohol was used as co-solvent. In current practice, this procedure, known as “alcohol-enhanced” Cu-mediated radiofluorination [34], has found wide application with various ArylBPin substrates. However, application of this procedure to [^18^F]SFB preparation (Scheme 5) with [^18^F]F^−^ elution from QMA carbonate cartridge (46 mg) by solution of Et_4_NHCO_3_ in *n*-BuOH and radiofluorination of **6** in DMA/*n*-BuOH (7/3, 110 °C, 20 min) resulted in a very low RCC of only 3% (entry 1, Table 3).

Therefore, we have switched to our previously developed [^18^F]fluoride trapping-elution procedure by replacing Et_4_NHCO_3_ with a non-basic PTC, Bu_4_NOTf, in conjunction with 2-PrOH (0.6 mL) as the elution solvent and co-solvent [23]. The radionuclide retained on the QMA carbonate cartridge (46 mg) was recovered with the solution of the PTC in 2-PrOH (82% recovery rate); the eluate was collected in a reaction vial pre-filled with Cu catalyst and **6** in CH_3_CN or DMA, followed by immediate heating to 65 °C for 10 min, and then the second round of heating to 110 °C for 15 min. Unfortunately, in both reaction solvents, the RCC was as low as 9% (entries 2, 3, Table 3), and thus did not support the beneficial effect of alcohol as a co-solvent in the radiofluorination of **6** [23,34]. The results of these screening experiments prompted us to attempt the radiofluorination of **6** under alcohol-free conditions. To recover the radionuclide retained on the QMA carbonate cartridge (46 mg), a solution of Bu_4_NOTf in MeOH was used giving a [^18^F]fluoride elution efficiency of 95%. The solvent was removed by heating under a stream of N_2_ gas. The Cu-catalyst and **6** dissolved in DMA were added to the dried residue and the labeling reaction was carried out at 65 °C for 10 min followed by an increase in temperature to 110 °C for a further 15 min. As a result we were able to increase the RCC values up to 49 and 57% for the precursor/copper catalyst ratios of 20/20 and 20/35 µmol, respectively (entries 4, 5, Table 3). Unfortunately, under these conditions, we observed losses of over 30% of radioactivity on the inner surfaces of the reaction vessel; these losses can be attributed to the absorption of the [^18^F]fluoride during the evaporation of MeOH. With the aim of developing a trapping-elution protocol for [^18^F]fluoride that does not use either water (to eliminate azeotropic drying) or alcohol, we attempted to use Oasis WAC 1cc (30 mg) as a weak-base anion exchange cartridge. This barrel-type cartridge was successfully used in our previous study [35] for the elution of the trapped [^18^F]fluoride with solution of a new PTC, 4-dimethylaminopyridinium trifluoromethane sulphonate (DMAPOTf), in DMI/DMA. Using this cartridge in the context of the current work, we have obtained a 93% recovery rate with a solution of Bu_4_NOTf (2.5 μmol) in DMA (0.8 mL). It should be emphasized that the elution step was carried out without resorting to the so-called “back-flushing” protocol (elution in the direction opposite to the load) which is not always compatible with modern synthesizers.

Fortunately, the radiofluorination step under previously established heating conditions and a precursor/copper catalyst ratio of 20/35 µmol resulted in an RCC of 56 ± 3% (entry 8, Table 3) coupled with a significant reduction in radioactivity loss on the inner surfaces of the reaction vessel (to 15% of total activity).

**Figure 1:**
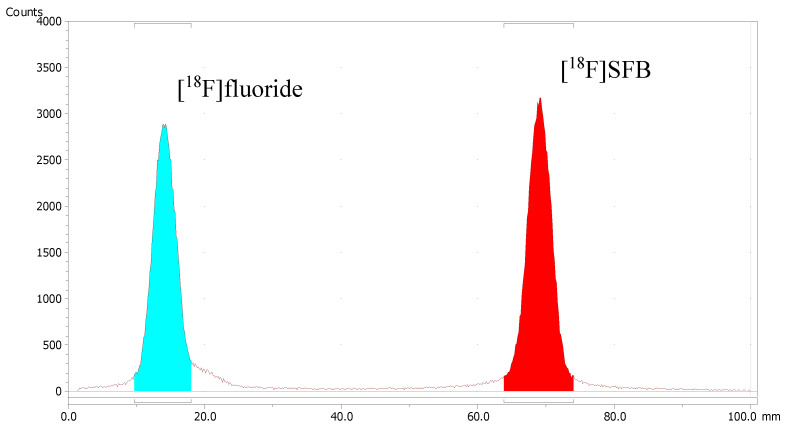
RadioTLC analysis of the reaction mixture for the synthesis of [^18^F]SFB from **6** (Scheme 5); silica plates, eluent ethyl acetate (TLC system 1); RCC 53% (entry 7, Table 3).

Having optimized the synthesis conditions, we proceeded to develop -the SPE-based purification of [^18^F]SFB using the remote-controlled synthesis module (in-house development). The design of the module is described in detail elsewhere [23,36]. According to the radioTLC analysis of the reaction mixture (Figure 1), the only radiochemical impurity was the unreacted [^18^F]fluoride, which can usually be removed using a suitable reverse-phase cartridge. The copper impurity species derived from the Cu(OTf)_2_(Py)_4_ catalyst can be removed using a cation-exchange cartridge. Using a combination of two cartridges connected in series, first a Sep-Pak Light Accell™ Plus CM Cartridge (CM cartridge) followed by an Oasis HLB 3cc Vac cartridge (60 mg), we were able to produce a radiochemically pure and copper-free solution of [^18^F]SFB in 1 mL of acetonitrile. The radiochemical purity of more than 99% and the identity of [^18^F]SFB were confirmed by HPLC (Figure S27). The residual copper content, as determined by ICP/MS, was 0.4 ± 0.2 ppm and was below any level of concern according to the ICH Guideline on Elemental Impurities (Q3D) [37]. It is worth noting that the proposed SPE procedure allowed for the complete removal of the unreacted precursor **6** as well as a potential by-product, 2,5-dioxopyrrolidin-1-yl benzoate (**2a**), which is often formed in the course of the Cu-mediated ^18^F-fluorodeborylation reactions [29]. HPLC analysis of the purified product co-injected with separately prepared **2a** (Scheme 2) showed that none of the impurity was present (Figure S28). This impurity is expected to compete with [^18^F]SFB in the peptide conjugation reaction thus reducing the “apparent” specific activity of the product. Overall, the developed one-step synthesis with SPE purification yielded [^18^F]SFB with a non-decay-corrected RCY of 30 ± 3% (n = 7) in about 35 min synthesis time and a molar activity in the range of 96–155 GBq/µmol.

### 2.3. Conjugation of [^18^F]SFB with Glutathione (GSH)

As a proof-of-concept, a conjugation reaction of the [^18^F]SFB with a model peptide, γ-glutamylcysteinylglycine (glutathione, GSH) was investigated using small aliquots (25 µL) of the purified [^18^F]SFB in acetonitrile. At GSH concentrations of 0.1 and 1 mg/mL (reaction time 30 min, phosphate buffer, pH of 8.4 [38]) the observed conjugation efficiency exceeded 99% according to HPLC data (Figure S29). However, it should be noted that aliquot labeling assays can lead to overestimation of labeling efficiency due to several factors such as reduced compatibility of the solvent used due to exceedingly small volumes, etc. It is therefore not excluded that for full-batch peptide labeling with [^18^F]SFB, the conditions for such labeling would need to be further optimized depending on the specific substrate.

## 3. Materials and Methods

### 3.1. General Chemistry

Melting points were measured using a melting point apparatus and are uncorrected. ^1^H (400.17 MHz) and ^13^C (100.63 MHz) spectra were recorded on a Bruker Avance III HD 400 NanoBay NMR spectrometer (Bruker BioSpin AG, Fällanden, Switzerland) in CDCl_3_ or dimethylsulfoxide (DMSO)-d_6_ ((Solvex, Moscow, Russia) at ambient temperature. All ^13^C NMR spectra were proton decoupled. The signal patterns are indicated as follows: s = singlet, d = doublet, dd = doublet of doublets, ddd = doublet of doublet of doublets, t = triplet, m = multiplet, br = broad. Integrals are given according to the assignments; coupling constants are given in Hertz. Unless otherwise stated, all reagents were purchased from commercial suppliers (Merck KGaA, Darmstadt, Germany) and used as received. γ-glutamylcysteinylglycine (glutathione, GSH) was purchased from Millipore Sigma (Taufkirchen, Germany). Solvents were dried by standard procedures and freshly distilled before use.

#### 3.1.1. The Three Step Synthesis of (4-((2,5-Dioxopyrrolidin-1-yloxy)carbonyl)phenyl)(phenyl)iodonium Trifluoroacetate (**4**) (Scheme 2)

2,5-Dioxopyrrolidin-1-yl benzoate (**2a**) was prepared analogously according to the method [39].

To the suspension of benzoic acid (**1a**) (1.22 g, 10 mmol) and di(*N*-succinimidyl) carbonate (2.56 g, 10 mmol) in dry acetonitrile (10 mL) at 10 °C, triethylamine (1.60 g, 16 mmol) was added in one portion. The resulting solution was stirred for 20 h at 20 °C. The solution was filtered to remove small impurities and evaporated in vacuo. The residue was dissolved in DCM (50 mL) and washed with saturated NaHCO_3_ (3 × 20 mL). The organic layer was dried (Na_2_SO_4_) and evaporated in vacuo. **2a** was obtained as a white solid after recrystallization from ethanol, yield 0.9 g (41%). m.p. 143–144 °C (ethanol). NMR spectra are consistent with the literature [39]. ^1^H NMR (400 MHz, CDCl_3_): δ = 8.11 (d, *J* = 8.0 Hz, 2H), 7.66 (t, *J* = 7.5 Hz, 1H), 7.49 (t, *J* = 7.6 Hz, 2H), 2.87 (s, 4H). ^13^C NMR (101 MHz, CDCl_3_): δ = 169.4 (two C=O), 161.9, 135.0, 130.6, 128.9, 125.1, 25.7.

2,5-Dioxopyrrolidin-1-yl 4-fluorobenzoate ([^19^F]SFB; **2b**).

From 4-fluorobenzoic acid **1b** (1.40 g, 10 mmol), the compound **2b** was prepared as a white solid, according to the above procedure, yield 1.05 g (44%). m.p. 119–120 °C (ethanol). The NMR spectra are consistent with the literature [18]. ^1^H NMR (400 MHz, CDCl_3_): δ = 8.15 (m, 2H), 7.18 (m, 2H), 2.89 (s, 4H). ^13^C NMR (101 MHz, CDCl3): δ = 169.4 (two C=O), 166.9 (d, *J* = 259.4 Hz), 161.0, 133.5 (d, *J* = 9.9 Hz), 121.4 (d, *J* = 3.0 Hz), 116.4 (d, *J* = 22.4 Hz), 25.8. ^19^F {^1^H} NMR (376.5 MHz, CDCl_3_): δ = −101.3.

2,5-Dioxopyrrolidin-1-yl 4-iodobenzoate (**2c**).

From 4-iodobenzoic acid **1c** (4.96 g, 20 mmol), compound **2c** was prepared as a white solid, according to the above procedure, yield 5.05 g (73%). m.p.—decomposed higher than 140 °C. The NMR spectra are consistent with the literature [40]. ^1^H NMR (400 MHz, CDCl_3_): δ = 8.02 (d, *J* = 8.4 Hz, 2H), 7.79 (d, *J* = 8.4 Hz, 2H), 2.85 (s, 4H). ^13^C NMR (101 MHz, CDCl_3_): δ = 170.2 (two C=O), 161.7, 138.6, 131.4, 123.9, 105.0, 25.6.

2,5-Dioxopyrrolidin-1-yl 4-(tri-*n*-butylstannyl)benzoate **3**.

*n*-Bu_6_Sn_2_ (6.38 g, 11.0 mmol) was added to the solution of 2,5-dioxo-1-pyrrolidinyl-4-iodobenzoate **2c** (1.90 g, 5.5 mmol) and Pd(PPh_3_)_4_ (0.20 g, 0.17 mmol, 3% mol) in dry toluene (35 mL) and dry DMF (35 mL) under an inert atmosphere. The mixture was refluxed 4 h, cooled to r.t. and the solvents were evaporated in vacuo. The residue was purified by column chromatography on silica gel (heptane/ethyl acetate = 9:1) to afford compound **3** (1.80 g, yield 62%) as a thick colorless oil [41]. ^1^H NMR (400 MHz, CDCl_3_): δ = 8.03 (d, *J* = 7.2 Hz, 2H), 7.62 (d, *J* = 7.2 Hz, 2H), 2.89 (s, 4H), 1.40–1.65 (m, 6H), 1.24–1.39 (m, 6H), 0.99–1.20 (m, 6H), 0.88 (t, *J* = 7.3 Hz, 9H). ^13^C NMR (101 MHz, CDCl_3_): δ = 169.5 (two C=O), 162.4, 153.3 (*J*_C-Sn_ = 326; 334 Hz), 136.9 (*J*_C-Sn_ = 30.0 Hz), 129.2 (*J*_C-Sn_ = 37.7 Hz), 124.5 (*J*_C-Sn_ = 9.3 Hz), 29.1 (*J*_C-Sn_ = 20.8 Hz), 27.4 (*J*_C-Sn_ = 55.6 Hz), 25.8, 13.8, 9.8 (*J*_C-Sn_ = 328; 343 Hz).

[4-(2,5-Dioxopyrrolidin-1-yloxycarbonyl)phenyl](phenyl)iodonium trifluoroacetate **4**.

(Bis(trifluoroacetoxy)iodo)benzene (1.20 g, 2.8 mmol) was added to the cooled (10 °C) solution of 2,5-dioxopyrrolidin-1-yl 4-(tri-*n*-butylstannyl)benzoate **3** (1.15 g, 2.3 mmol) in DCM (15 mL). After stirring at 20 °C for 40 h, the solution was evaporated in vacuo. The residue was purified by column chromatography on silica gel (DCM /methanol = 100:0 to 98:2) to afford compound **4** (0.35 g, yield 28%) as a white solid [42]. ^1^H NMR (400 MHz, DMSO-d_6_): δ = 8.47 (d, *J* = 8.6 Hz, 2H), 8.29 (d, *J* = 8.2 Hz, 2H), 8.18 (d, *J* = 8.6 Hz, 2H), 7.69 (t, *J* = 7.5 Hz, 1H), 7.55 (d, *J* = 7.8 Hz, 2H), 2.89 (s, 4H). ^13^C NMR (101 MHz, DMSO-d_6_): δ = 170.1, 161.0, 136.1, 135.4, 132.6, 132.3, 131.9, 127.3, 123.8, 116.9, 25.6. ^19^F {^1^H} NMR (376.5 MHz, CDCl_3_): δ = −73.4.

#### 3.1.2. One-Step Synthesis of the ArylBPin Precursor—2,5-Dioxopyrrolidin-1-yl 4-(4,4,5,5-tetramethyl-1,3,2-dioxaborolan-2-yl)benzoate **6** (Scheme 4)

Triethylamine (1.60 g, 16 mmol) was added in one portion to the suspension of 4-(4,4,5,5-tetramethyl-1,3,2-dioxaborolan-2-yl)benzoic acid **5** (2.48 g, 10 mmol) and di(*N*-succinimidyl) carbonate (2.56 g, 10 mmol) in dry acetonitrile (10 mL) at 10 °C. The resulting solution was stirred for 20 h at 20 °C. The solution was filtered to remove the small impurities and evaporated in vacuo. The residue was dissolved in DCM (50 mL) and washed with saturated NaHCO_3_ (3 × 20 mL). The organic layer was dried (Na_2_SO_4_) and evaporated to give 4-(4,4,5,5-tetramethyl-1,3,2-dioxaborolan-2-yl)benzoic acid *N*-succinimidyl ester **6** without the need of further purification, yield 1.75 g (51%), white solid. m.p. 192–193 °C (acetone-hexane 1:1). The NMR spectra are consistent with the literature [18]. ^1^H NMR (400 MHz, CDCl_3_): δ = 8.10 (d, *J* = 8.1 Hz, 2H), 7.92 (d, *J* = 8.1 Hz, 2H), 2.90 (s, 4H), 1.36 (s, 12H). ^13^C NMR (101 MHz, CDCl_3_): δ = 169.3 (two C=O), 162.1, 135.1, 129.6, 127.3, 84.6, 25.8, 25.0. ^1^H NMR (400 MHz, CDCl_3_): δ = 8.10 (d, *J* = 8.1 Hz, 2H), 7.92 (d, *J* = 8.1 Hz, 2H), 2.90 (s, 4H), 1.36 (s, 12H). ^13^C NMR (101 MHz, CDCl_3_): δ = 169.3 (two C=O), 162.1, 135.1, 129.6, 127.3, 84.6, 25.8, 25.0. Carbon adjacent to boron was not observed.

#### 3.1.3. The 2 Step Synthesis of L-γ-Glutamyl-S-(4-fluorobenzoyl)-L-cysteinylglycine ([^19^F]SFB-GSH) (Scheme S1)

1H-Benzotriazol-1-yl(4-fluorophenyl)methanone **7** [43].

A solution of 1H-benzotriazole (7.91 g, 66.4 mmol) and SOCl_2_ (2.80 g, 23.5 mmol) in DCM (30 mL) was added dropwise to a suspension of 4-fluorobenzoic acid (**1b**) (3.00 g, 21.4 mmol) in DCM (20 mL) over 5 min. After stirring at 20 °C for 2 h, the mixture was filtered, the solid (BtH × HCl) was washed with DCM (2 × 7 mL) and discarded. The solution was evaporated in vacuo and the residue was washed with warm Et2O to give compound **7** as a white solid (4.10 g, 79%). The NMR spectra are consistent with [43]. ^1^H NMR (400 MHz, CDCl_3_): δ = 8.38 (ddd, *J* = 8.3 Hz, 1H), 8.34–8.29 (m, 2H), 8.17 (ddd, *J* = 8.3 Hz, 1H), 7.71 (ddd, *J* = 8.3 Hz,7.1, 1.0 Hz, 1H), 7.55 (ddd, *J* = 8.3 Hz,7.1, 1.0 Hz, 1H), 7.29–7.23 (m, 2H). ^13^C NMR (101 MHz, CDCl_3_): δ = 166.7 (d, *J*_CF_ = 256.4 Hz), 165.5, 145.9, 134.8 (d, *J*_CF_ = 9.5 Hz), 132.5, 130.6, 127.7 (d, *J*_CF_ = 3.3 Hz), 126.6, 120.4, 115.9 (d, *J*_CF_ = 22.0 Hz), 114.9. ^19^F {^1^H} NMR (376.5 MHz, CDCl_3_): δ = −103.4.

L-γ-Glutamyl-S-(4-fluorobenzoyl)-L-cysteinylglycine ([^19^F]SFB-GSH) was prepared according to the method [44].

The solution of glutathione (0.62 g, 2.0 mmol) in water (11 mL) was added immediately with stirring to the solution of 1H-benzotriazol-1-yl(4-fluorophenyl)methanone (**7**) (0.48 g, 2.0 mmol) in methanol (40 mL). To the resulting suspension, a solution of KHCO_3_ (0.40 g, 4.0 mmol) in water (5 mL) was added dropwise during 5 min. The clear solution was stirred at 20 °C for 20 min and then aq. 4M HCl was added dropwise to pH 5. The resulting suspension was filtered and the solid was washed with water (3 × 10 mL) and acetone (3 × 10 mL). Drying in vacuo yielded compound (0.66 g, 77%) as a white solid, m.p. 254–256 °C. ^1^H NMR (400 MHz, DMSO-d6), low solubility, part of the signals have been seen: δ = 8.76 (br.s, 1H, NH), 8.57 (br.d, 1H, NH), 7.90–8.07 (m, 2H, 2CH^Ar^), 7.38 (m, 2H, 2CH^Ar^). ^19^F {^1^H} NMR (376.5 MHz, DMSO-d_6_): δ = −104.8. ^1^H NMR (400 MHz, DMSO-d_6_+TFA): δ = 8.50–8.38 (m, ~5H) and 8.28 (br.s, ~5H), 7.98 (m, 2H), 7.39 (t, *J* = 8.7 Hz, 2H), 4.59 (m, 1H), 3.93 (br.s, 1H), 3.79 and 3.74 (two dd, *J* = 5.9; 17.5 Hz, 2H), 3.51 (dd, *J* = 4.9; 13.5 Hz, 1H), 3.20 (dd, *J* = 8.8; 13.5 Hz, 1H), 2.24–2.43 (m, 2H), 1.90–2.10 (m, 2H). ^13^C NMR (101 MHz, DMSO-d_6_+TFA): δ = 189.4 (C(O)S), 171.3 (C), 171.0 (C), 170.9 (C), 170.1 (C), 165.5 (d, *J*^1^_CF_ = 252.4 Hz, CF), 133.0 (d, *J*^4^_CF_ = 2.8 Hz, C), 130.0 (d, *J*^3^_CF_ = 9.6 Hz, 2CH^Ar^), 116.3 (d, *J*^2^_CF_ = 22.2 Hz, 2CH^Ar^), 51.8 (CH), 51.6 (CHS), 41.0 (CH_2_), 31.0 (CH_2_), 30.7 (CH_2_), 26.1 (CH_2_). ^19^F {^1^H} NMR (376.5 MHz, DMSO-d_6_+TFA): δ = −104.8.

### 3.2. Radiochemistry

#### 3.2.1. General

Unless otherwise stated, reagents and solvents were of commercial quality and were used without further purification. The precursors 4, 6, the reference standard [^19^F]SFB (**2a**), and the reference standard for the conjugate of [^19^F]SFB with glutathione—[^19^F]SFB-GSH—were prepared as described above. Anhydrous acid-free CH_3_CN (max. 10 ppm H_2_O) was purchased from Kriochim, St. Petersburg, Russia. Deionized water (18.2 MΩ) from an in-house Millipore Simplicity purification system was used for the preparation of all aqueous solutions. [^18^O]H_2_O (97% enrichment) was purchased from Global Scientific Technologies (Sosnovy Bor, Russia). Cu(OTf)_2_(Py)_4_ was purchased from Sigma-Aldrich GmbH (Steinheim, Germany) and stored under argon. All the cartridges used were purchased from Waters Corporation (Millford, CT, USA). Prior to use, the Sep-Pak Accell Plus QMA Plus Light cartridge (130 mg) was pre-treated with 10 mL of 0.5 M NaHCO_3_ followed by 10 mL of water. The Sep-Pak Accel™ Plus QMA Carbonate cartridge (46 mg) was pre-treated with 10 mL of 0.05 M NaHCO_3_ followed by 10 mL of water. The Sep-Pak Accell Plus CM Plus Light cartridge (360 mg) was rinsed with 3 mL of water. Oasis WAX 1 cc Vac Cartridge, 30 mg sorbent per cartridge, 60 µm, and Oasis HLB 3 cc Vac cartridge, 60 mg sorbent per cartridge, 30 µm, were not conditioned. All the radiosyntheses were carried out on a remote-controlled synthesis module (in-house development); the reactions were performed in 5 mL V-shaped vials with a screw cap (Wheaton) placed into heating block, with magnetic stirring, and the transfer of all the reagents was achieved through nitrogen flow. The module design is described in detail elsewhere [23,36]. Radioactivity losses on the inner surface of the reaction vessel were evaluated by measuring the activity of the reaction vessel with reaction mixture and after emptying the reaction vessel (the contents were aspirated with a syringe) and rinsing the reactor with water.

The analytical HPLC system consisted of a Dionex ISC-5000 gradient pump (Dionex, Sunnyvale, CA, USA), a Rheodyne type injector with a 20 μL loop, and a Dionex VWD variable wavelength absorbance detector (set at 254 nm) connected in series with a radio detector (Carrol and Ramsey Associates, CA, USA, model 105-S) giving a delay of 0.1 min. Two different HPLC set-ups were used. The identity and radiochemical purity of [^18^F]SFB was determined using the following HPLC method (HPLC system 1): XBridge C18 HPLC column, 150 × 4.6 mm (Waters Corporation, Milford, CT, USA), eluent: mixture of water and acetonitrile using gradient elution conditions, flow rate 2.0 mL/min. Gradient: 0.0–8.0 min 5–95% CH_3_CN linear increase; 8.0–8.2 min 95–5% CH_3_CN linear decrease; 8.2–12.0 min 5% CH_3_CN isocratic (column equilibration). The R_t_ values for reference standard [^19^F]SFB and [^18^F]SFB were 4.8 and 4.9 min, respectively. The same HPLC method was used to monitor the course of the radiofluorination reaction.

The identity of the [^18^F]SFB-GSH conjugate was confirmed by co-injection with the reference standard [^19^F]SFB-GSH using the same HPLC column (XBridge C18 HPLC column, 150 × 4.6 mm) under the following conditions (HPLC system 2): eluent: mixture of an aqueous solution of Na_2_HPO_4_ 10 mM, pH of 8.5 (adjusted with H_3_PO_4_) with acetonitrile under gradient elution conditions, flow rate 2.0 mL/min. Gradient: 0–2.0 min 0% CH_3_CN isocratic; 2.0–10.0 min 0–95% CH_3_CN linear increase; 10.0–10.2 min 95–0% CH_3_CN linear decrease; 10.2–14.0 min 0% CH_3_CN isocratic (column equilibration). The R_t_ values for the reference standard [^19^F]SFB, [^18^F]SFB, [^19^F]SFB-GSH and [^18^F]SFB-GSH were 7.1, 7.2, 5.0, and 5.1 min, respectively. The same HPLC conditions were used to evaluate the conjugation efficiency.

Radio-TLC analysis was performed using a Scan-RAM radio-TLC scanner controlled by the chromatography software package Laura for PET (LabLogic, Sheffield, UK). After an aliquot of 2–3 μL of the reaction mixture was applied to a TLC plate, the plate was developed using one of the set-ups described below. To follow the course of the radiofluorination reaction, the TLC system 1 was used, as follows: pre-coated silica gel 60 F254 plates (Sorbfil, Lenchrom Ltd., St. Petersburg, Russia) were eluted with ethyl acetate. The R_f_ values for [^18^F]fluoride and [^18^F]SFB were 0.05 and 0.49, correspondingly. To evaluate the conjugation rate of [^18^F]SFB with glutathione (GSH), TLC analysis was performed on the silica gel 60 RP-18 F254 plates, aluminum sheets (Merck, Darmstadt, Germany), eluent acetonitrile/water (50/50) (TLC system 2). R_f_ values for [^18^F]SFB and [^18^F]SFB-GSH were 0.45 and 0.95, respectively. Radiochemical conversion (RCC) measured by radio-TLC was defined as the ratio of the product peak area to the total peak area on the TLC. RCC values were not corrected for radioactive decay. The copper content in the final formulation was determined using a Varian 725-ES ICP Optical Emission Spectrometer, (Spectralab Scientific Inc., Markham, ON, Canada).

#### 3.2.2. Production of [^18^F]Fluoride

[^18^F]Fluoride was produced via the ^18^O(p,n)^18^F nuclear reaction by irradiating [^18^O]H_2_O (97% enrichment, Global Scientific Technologies, Sosnovy Bor, Russia) in a silver target (1.2 mL) with 16.4 MeV protons in a PETtrace 4 cyclotron (GE Healthcare, Uppsala, Sweden). The irradiated [^18^O]H_2_O was transferred from the target into the collection vial using a helium flow.

#### 3.2.3. Synthesis of [^18^F]SFB

All reactions were carried out using a remote-controlled synthesis module (in-house development):

Method A (Scheme 2). The radiofluorination of (4-((2,5-dioxopyrrolidin-1-yloxy)carbonyl)phenyl)(phenyl)iodonium trifluoroacetate (**4**).

A solution of [^18^F]F^−^ (0.5–1.0 GBq) in [^18^O]H_2_O was loaded onto an Oasis Wax 1cc cartridge. The cartridge was rinsed with MeOH (2 mL) and dried under N_2_ gas flow for 2 min. [^18^F]F^−^ was eluted from the cartridge with a solution of **4** (10 mg, 18 μmol) in MeOH (0.2 mL)/CH_3_CN (0.5 mL) into the 5 mL V-vial reaction vessel. The reaction mixture was heated to 75 °C for 10 min, followed by a second round of heating to 120 °C for 15 min. At the end of the heating cycle, the reaction vessel was cooled to 40 °C.

Method B (Scheme 4). Copper-mediated radiofluorination of 2,5-dioxopyrrolidin-1-yl 4-(4,4,5,5-tetramethyl-1,3,2-dioxaborolan-2-yl)benzoate (**6**).

A solution of [^18^F]F^−^ (10–11 GBq) in [^18^O]H_2_O was loaded onto an Oasis Wax 1cc cartridge. The cartridge was flushed with MeOH (2 mL) and dried under N_2_ gas flow for 2 min. The trapped [^18^F]fluoride was then eluted with a solution of 0.9–1.2 mg of Bu_4_NOTf in 0.8 mL of DMA and the eluate was collected into the reaction vessel prefilled with 7 mg (20 µmol) of **6** and 23 mg (35 µmol) of Cu(OTf)_2_(Py)_4_. The reaction mixture was heated to 65 °C for 10 min, followed by a second round of heating to 110 °C for 15 min with stirring. At the end of the heating cycle, the reaction vessel was cooled to 40 °C.

#### 3.2.4. SPE Purification of [^18^F]SFB

The crude reaction mixture was diluted with 9 mL of H_2_O under stirring. The resulting solution was passed through a Sep-Pak Accell Plus CM Plus Light cartridge (360 mg) connected in series with an Oasis HLB 3 cc Vac cartridge (60 mg) followed by 7 mL of 5% CH_3_CN. The trapped [^18^F]SFB was then eluted with 1 mL of CH_3_CN.

#### 3.2.5. Conjugation of [^18^F]SFB with Glutathione (GSH)

An aliquot of a solution of the SPE-purified [^18^F]SFB (25 µL) was added to a GSH solution (0.1 or 1 mg/mL) in 0.9 mL of NaH_2_PO_4_ buffer (pH 8.4). The conjugation reaction was carried out at 28 °C for 30 min. An aliquot of the reaction mixture was analyzed by radio-HPLC (System 2) to evaluate the conjugation rate.

## 4. Conclusions

In the present work, two approaches for the direct labeling of [^18^F]SFB, a well-known fluorine-18 labeling prosthetic group, were evaluated, starting from diaryliodonium (DAI) salt **4** and pinacol aryl boronate **6** precursors. Compared to the DAI salt, the Cu-mediated ^18^F-fluorination of the pinacol aryl boronate precursor gave a higher RCC of 56 ± 3% (n = 7), making this approach more attractive for the development of a one-pot synthesis procedure of the [^18^F]SFB. Following radiofluorination in the presence of Bu_4_NOTf as a phase transfer catalyst, product isolation was achieved by a rapid and simple SPE-based purification procedure using a combination of a cation-exchange cartridge (Sep-Pak Light Accell™ Plus CM) connected in a series with an OASIS HLB 3 cc cartridge. In a full-batch synthesis, [^18^F]SFB was obtained at a non-decay-corrected RCY of 30 ± 3% (n = 7) with a radiochemical purity exceeding 99% and a molar activity in the range of 96–155 GBq/µmol, with synthesis time not exceeding 35 min. The developed cartridge-based purification procedure allowed the removal not only of residual copper, but also of the unreacted labeling precursor **6,** as well as potentially problematic unlabeled by-products that may be formed in the course of Cu-mediated [^18^F]fluorodeborylation. As a proof of concept, a conjugation reaction of the [^18^F]SFB with a model peptide, glutathione (GSH), was performed using small aliquots (25 µL) of the purified [^18^F]SFB with an observed labeling efficiency of over 99%. Compared to other recently reported one-pot [^18^F]SFB syntheses based on a Cu-catalyzed approach with final HPLC purification of the product, the presented method offers significant advantages in terms of simplicity and greatly reduced synthesis time; the latter is particularly important considering that the labeled prosthetic group thus obtained will be further used for the in-multistep radiolabeling of peptides.

## Figures and Tables

**Table 1:** Radiofluorination of **4** under different conditions; eluent: 18 µmol of **4** in 0.7 mL of the solvent; RCC—radiochemical conversion of **4** to [^18^F]SFB as calculated from radioTLC data.

Entry	Eluent	Radiofluorination		RCC, %
		Solvent	T, °C/t, min	
1	**4** (18 µmol) in MeOH/PC (0.2/0.5 mL)	MeOH/PC	(1) 75/10 (2) 120/20	5
2	**4** (18 µmol) in 2-PrOH/PC (0.5/0.5 mL)	2-PrOH/PC	(1) 85/10 (2) 120/20	1
3	**4** (18 µmol) in MeOH/diglim (0.2/0.5 mL)	MeOH/diglim	(1) 75/10 (2) 150/15	0
4	**4** (18 µmol) in MeOH/CH_3_CN (0.2/0.5 mL)	MeOH/CH_3_CN	(1) 75/10 (2) 120/15	36

**Table 2:** Comparison of published one-step syntheses and purification methods for [^18^F]SFB.

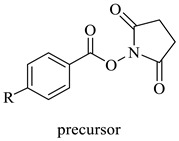						
R	Radiofluorination		Purification	Automation/t, min	Decay-Corrected RCY, %	Reference
	PTC	Solvent/T, °C/t, min				
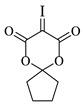	K_2.2.2._/K_2_CO_3_	DMF/130/4	HPLC	n.a./60	3–22 *	[17]
Bu_3_Sn-	Et_4_NOTf	DMA/140/10 Cu(OTf)_2_(Py)_4_	SPE + HPLC	Neptis/n.a.	42 ± 4 (n = 3)	[20]
PinB-	KOTf/K_2_CO_3_	DMI/90/15 Cu(OTf)_2_(Py)_4_	HPLC	TRACERLab FX 2N/ 68–83	22–34 * (n = 6)	[19]
	Bu_4_NOTf	DMA/65/10–110/15 Cu(OTf)_2_(Py)_4_	SPE	Remote-controlled/35	38 ± 3 * (n = 7)	This work

* Decay-corrected RCYs values were calculated based on the reported non-decay-corrected values with respect to the synthesis time; n.a.—not available.

**Table 3:** The results of the Cu-mediated radiofluorination of **6**: [^18^F]fluoride elution from the cartridge by solution of the PTC (2.5–10 µmol) in a suitable solvent; fluorination: 20–60 µmol of **6**, 20–35 µmol of Cu(OTf)_2_(Py)_4_ in 0.8 mL of reaction solvent; no access to atmospheric air; 65 °C, 10 min followed by 110 °C, 15 min; RCC—radiochemical conversion based on radioTLC data.

Entry	Anion Exchange Cartridge	Eluent	Eluent Removal	6, μmol	Cu-Catalyst, μmol	Radio Fluorination Solvent	RCC, % (TLC)	Losses on the Reactor Walls %
1	QMA carbonate, 46 mg	Et_4_NHCO_3_ (10 μmol)/ *n*-BuOH (0.4 mL)	-	60	26.5	*n*-BuOH/DMA *	3	-
2		Bu_4_NOTf (10 μmol)/ 2-PrOH (0.6 mL)	-	20	20	2-PrOH/CH_3_CN	9	-
3			-		20	2-PrOH/DMA	9	-
4		Bu_4_NOTf (10 μmol)/ MeOH (0.8 mL)	+		20	DMA	49	30
5					35		57	33
6	WAX 1cc 30 mg	Bu_4_NOTf (2.5 μmol)/ DMA (0.8 mL)	-		20	DMA	42	25
7					30		53; 53	13; 22
8					35		56 ± 3 (n = 7)	15 ± 5 (n = 7)

* Fluorination 110 °C, 20 min.

## Data Availability

The data presented in this study are available on request from the corresponding author.

## Supplementary Materials

The following supporting information can be downloaded at: https://www.mdpi.com/article/10.3390/ph17121723/s1**.** Block I: NMR spectra. Figure S1: ^1^H NMR spectrum for compound **2a** (400 MHz, CDCl_3_); Figure S2: ^13^C NMR spectrum for compound **2a** (101 MHz, CDCl_3_); Figure S3: ^1^H NMR spectrum for compound **2b** (400 MHz, CDCl_3_); Figure S4: ^13^C NMR spectrum for compound **2b** (101 MHz, CDCl_3_); Figure S5: ^19^F NMR spectrum for compound **2b** (376.5 MHz, CDCl_3_); Figure S6: ^1^H NMR spectrum for compound **2c** (400 MHz, CDCl_3_); Figure S7: ^13^C NMR spectrum for compound **2c** (101 MHz, CDCl_3_); Figure S8: ^1^H NMR spectrum for compound **3** (400 MHz, CDCl_3_); Figure S9: ^13^C NMR spectrum for compound **3** (101 MHz, CDCl_3_); Figure S10: ^1^H NMR spectrum for compound **4** (400 MHz, DMSO-d_6_); Figure S11: ^13^C NMR spectrum for compound **4** (101 MHz, DMSO-d_6_); Figure S12: ^19^F NMR spectrum for compound **4** (376.5 MHz, DMSO-d_6_); Figure S13: ^1^H NMR spectrum for compound **6** (400 MHz, CDCl_3_); Figure S14: ^13^C NMR spectrum for compound **6** (101 MHz, CDCl_3_); Figure S15: ^1^H NMR spectrum for compound **7** (400 MHz, CDCl_3_); Figure S16: ^13^C NMR spectrum for compound **7** (101 MHz, CDCl_3_); Figure S17: ^19^F NMR spectrum for compound **7** (376.5 MHz, CDCl_3_); Figure S18: ^1^H NMR spectrum for compound ([^19^F]SFB-GSH) (400 MHz, DMSO-d_6_); Figure S19: ^19^F NMR spectrum for compound ([^19^F]SFB-GSH) (376.5 MHz, DMSO-d_6_); Figure S20: ^1^H NMR spectrum for compound ([^19^F]SFB-GSH) (400 MHz, DMSO-d_6_+TFA); Figure S21: ^13^C NMR spectrum for compound ([^19^F]SFB-GSH) (101 MHz, DMSO-d_6_+TFA); Figure S22: DEPT-135 NMR spectrum for compound ([^19^F]SFB-GSH) (101 MHz, DMSO-d_6_+TFA); Figure S23: ^19^F NMR spectrum for compound ([^19^F]SFB-GSH) (376.5 MHz, DMSO-d_6_+TFA); Figure S24: HSQC NMR spectrum for compound ([^19^F]SFB-GSH) (400 and 101 MHz, DMSO-d_6_+TFA); Figure S25: HMBC NMR spectrum for compound ([^19^F]SFB-GSH) (400 and 101 MHz, DMSO-d_6_+TFA). Block II. Radio-HPLC chromatograms. Figure S26: HPLC analysis (UV trace) of the methanolic solution of DAI precursor **4**: (A) before elution of [^18^F]fluoride from the cartridge; (B) after passing through the cartridge QMA carbonate cartridge. (46 mg); (C) after passing through Oasis WAX 1cc cartridge; HPLC column XBridge C18, 150 × 4.6 mm (Waters Corporation, Millford, CT, USA), eluent: 0.1% trifluoroacetic acid/acetonitrile (95/5, *v*/*v*), flow rate 2.0 mL/min, UV 254 nm; Figure S27: HPLC analysis of [^18^F]SFB (gradient conditions, HPLC system 1, UV 254 nm). (A) UV chromatogram of the authentic reference [^19^F]SFB; (B) radio-HPLC chromatogram of an aliquot of reaction mixture after radiofluorination; (C) radio-HPLC chromatogram of [^18^F]SFB after purification on the cartridges CM light and OASIS HLB 3cc; Figure S28: HPLC analysis (UV trace) of the [^18^F]SFB for the presence of the residual amounts of **6** and potential by-product **2a**; (A) authentic reference **6**; (B) authentic reference **2a**; (C) reaction mixture after radiofluorination of **6**; (D) [^18^F]SFB after purification on the cartridges CM light and OASIS HLB 3cc. HPLC system 1, UV 254 nm, gradient conditions; Figure S29: HPLC analysis of the [^18^F]SFB-GSH conjugate (gradient conditions, HPLC system 2, UV 254 nm). (A) UV trace of the authentic reference [^19^F]SFB (**2b**); (B) UV trace of the authentic reference [^19^F]SFB-GSH; (C) radio-HPLC chromatogram of the reaction mixture for [^18^F]SFB-GSH, >99% conjugation rate. Scheme S1. Synthesis of [^19^F]SFB-GSH. Reaction conditions. *v*: 1H-benzotriazole, SOCl_2_, DCM, 20 °C, 2 h; *vi*: glutathione, KHCO_3_, MeOH-H_2_O, 20 °C, 20 min.
