# Supplementary material for: Simple and Efficient Synthesis of N-Succinimidyl-4-[18F]fluorobenzoate ([18F]SFB)—An Important Intermediate for the Introduction of Fluorine-18 into Complex Bioactive Compounds
Source: Pharmaceuticals (Basel). 2024 Dec 20;17(12):1723. doi: 10.3390/ph17121723 (PMC11677304; doi:10.3390/ph17121723)
Supplement: Supplementary file 1 [file pharmaceuticals-17-01723-s001.zip › pharmaceuticals-3357531-supplementary.pdf]

Supporting information to

Simple and Efficient Synthesis of *N*-Succinimidyl-4-[^{18}F]fluorobenzoate ([^{18}F]SFB)—An Important Intermediate for the Introduction of Fluorine-18 into Complex Bioactive Compounds

Viktoriya V. Orlovskaya [1], Olga S. Fedorova [1], Nikolai B. Viktorov [2], Raisa N. Krasikova [1,*]

[1] *N. P. Bechtereva Institute of the Human Brain, Russian Academy of Sciences, 197022 St. Petersburg, Russia*

[2] *Faculty of Chemical and Biotechnology, St. Petersburg State Institute of Technology (Technical University), 190013 St. Petersburg, Russia*

Block I. NMR spectra

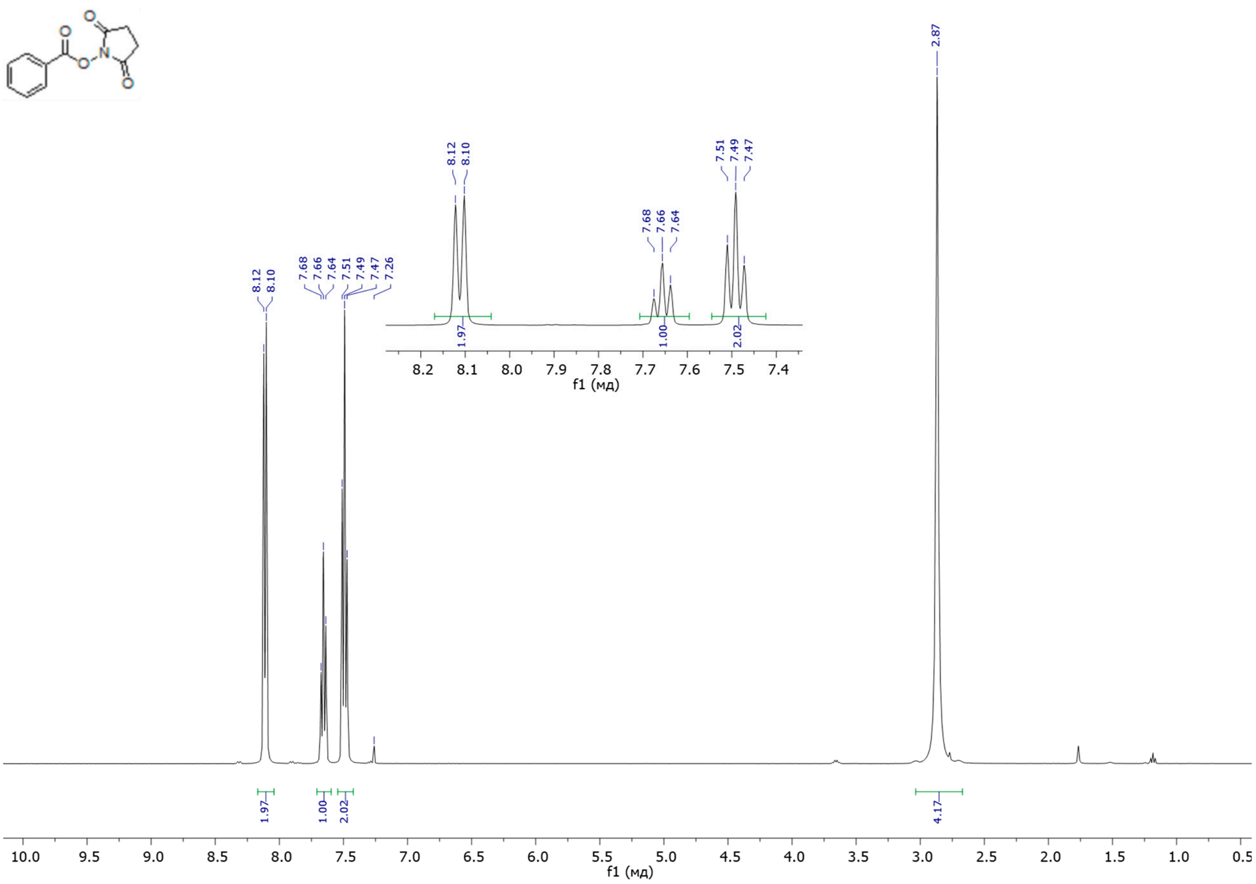

Figure S1. ^{1}H NMR spectrum for compound **2a** (400 MHz, $CDCl_3$).

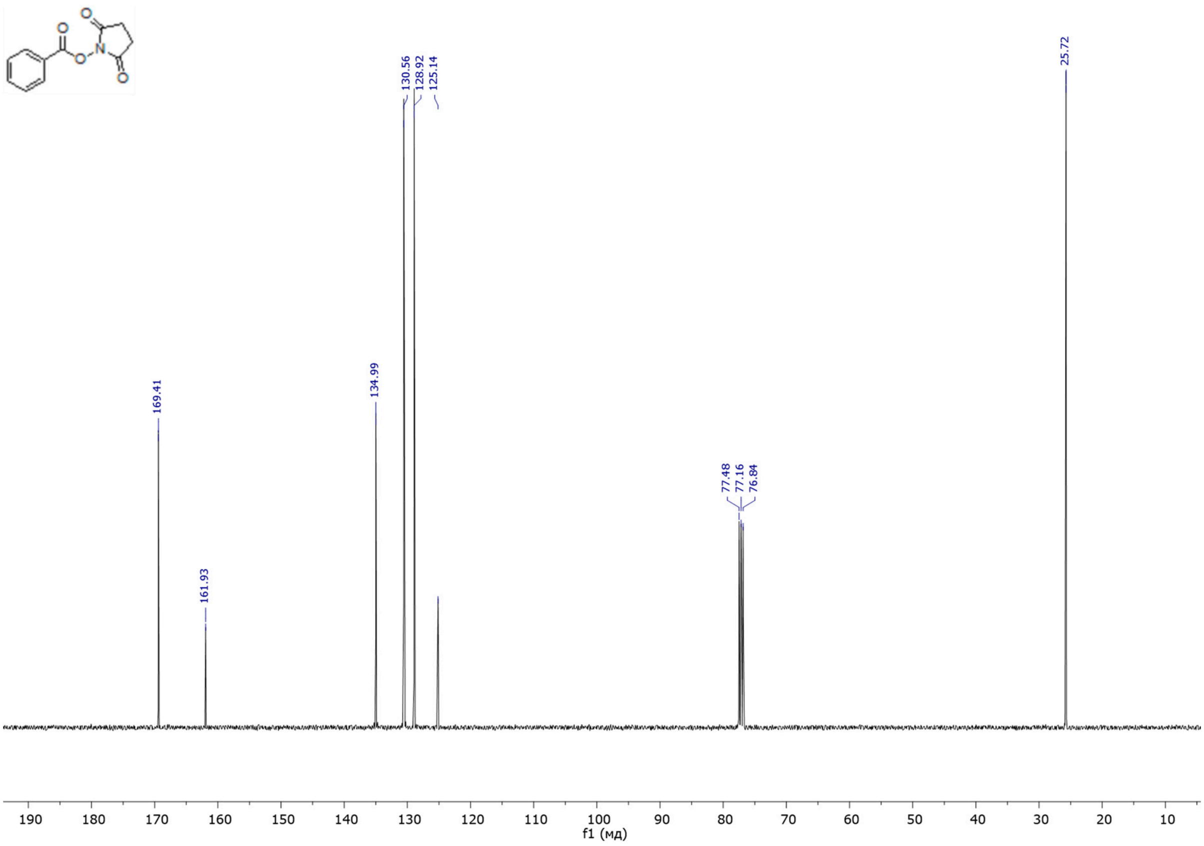

Figure S2. ^{13}C NMR spectrum for compound **2a** (101 MHz, $CDCl_3$).

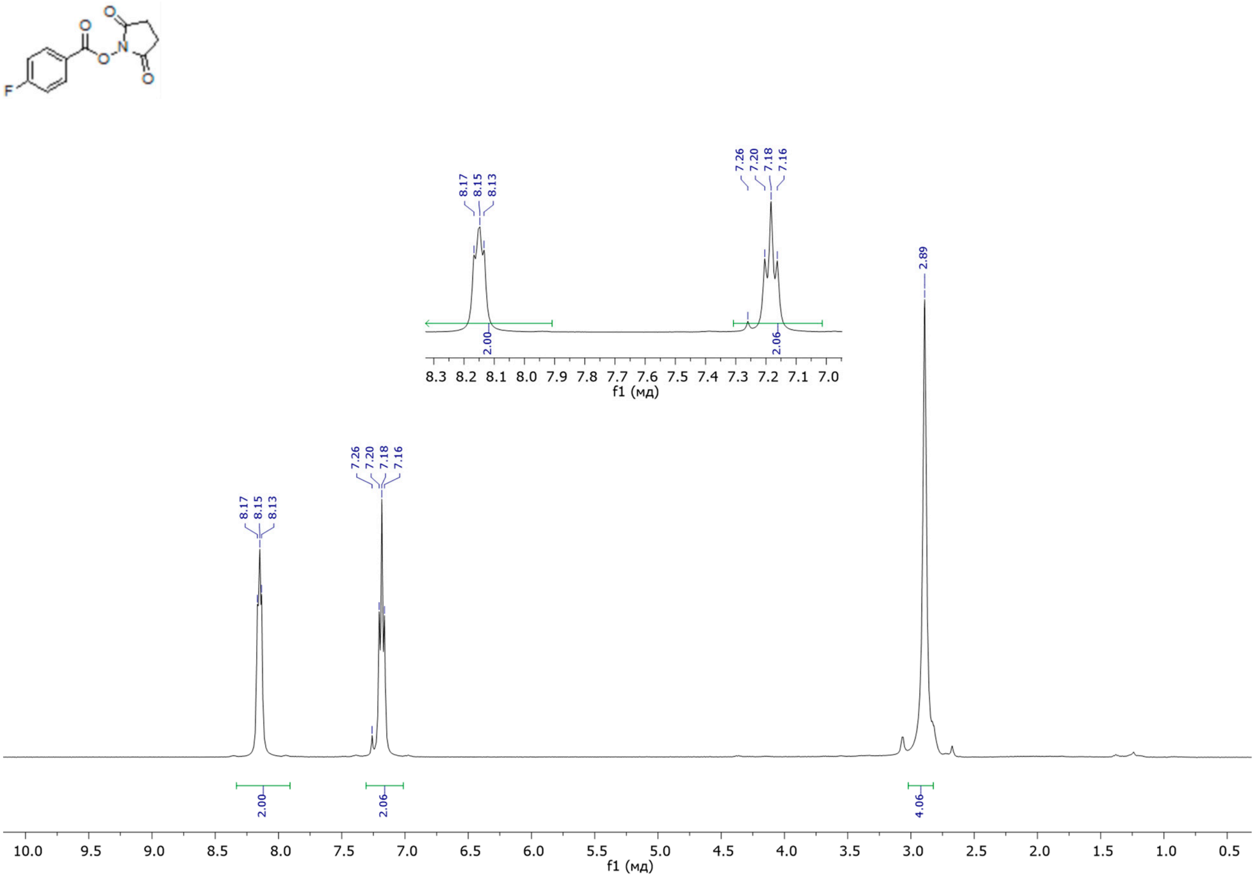

Figure S3. ^{1}H NMR spectrum for compound **2b** (400 MHz, $CDCl_3$).

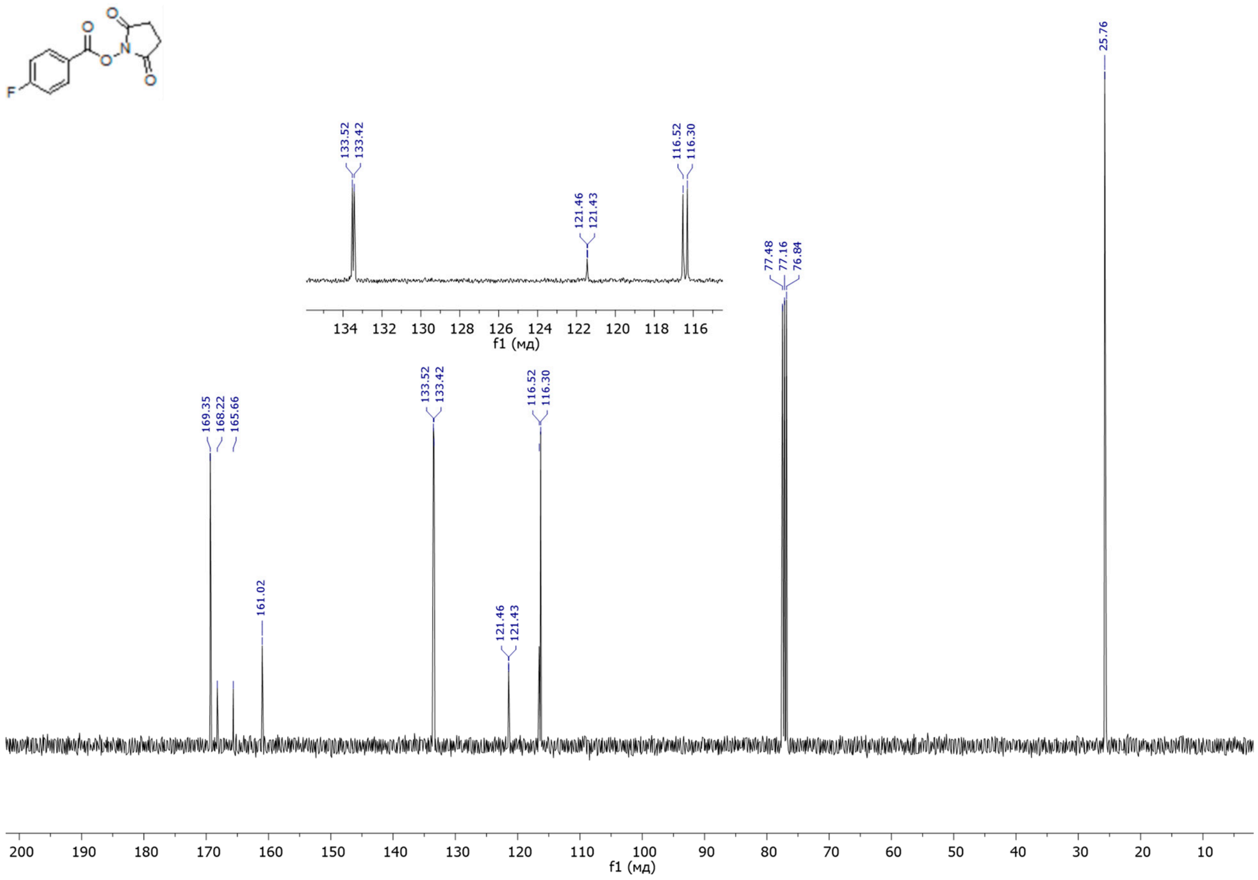

Figure S4. ^{13}C NMR spectrum for compound **2b** (101 MHz, $CDCl_3$).

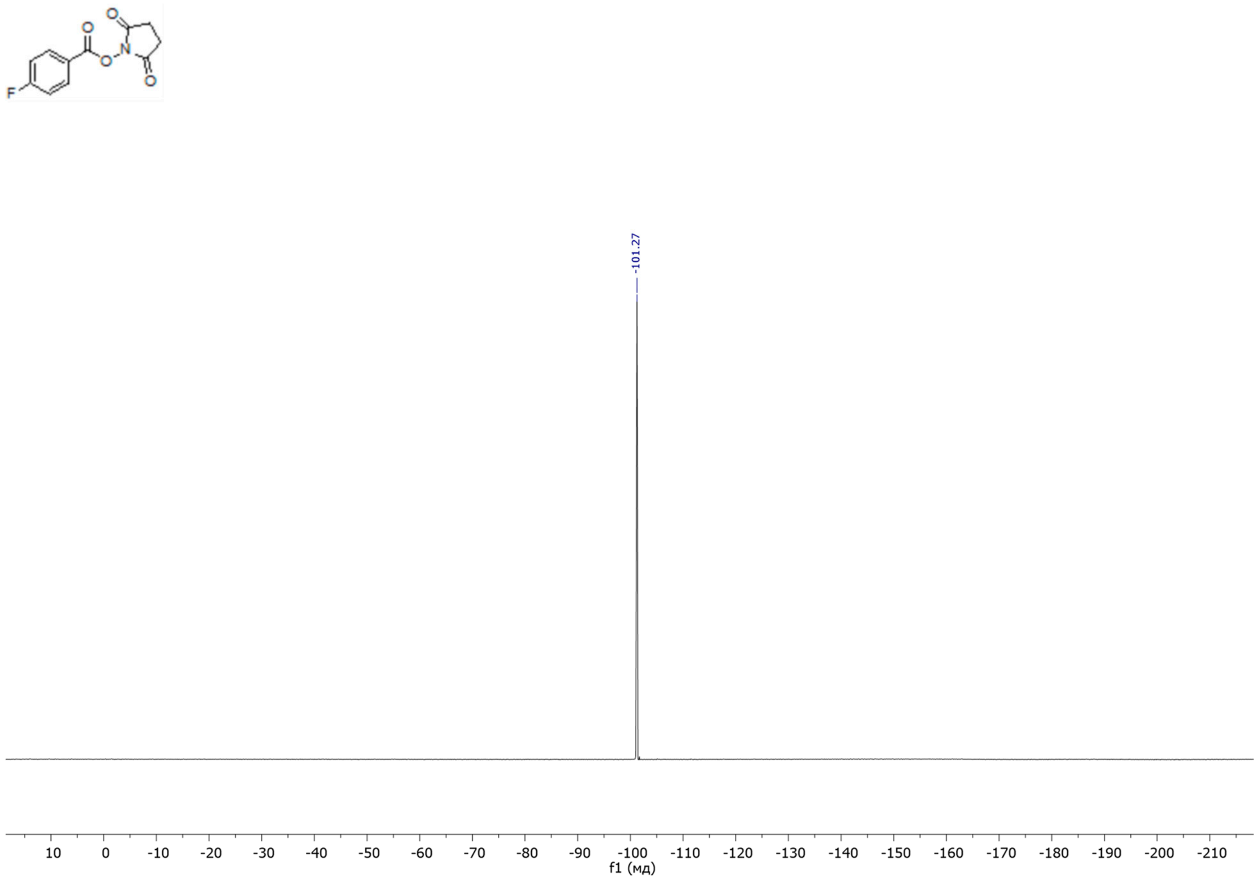

Figure S5. ^{19}F NMR spectrum for compound **2b** (376.5 MHz, $CDCl_3$).

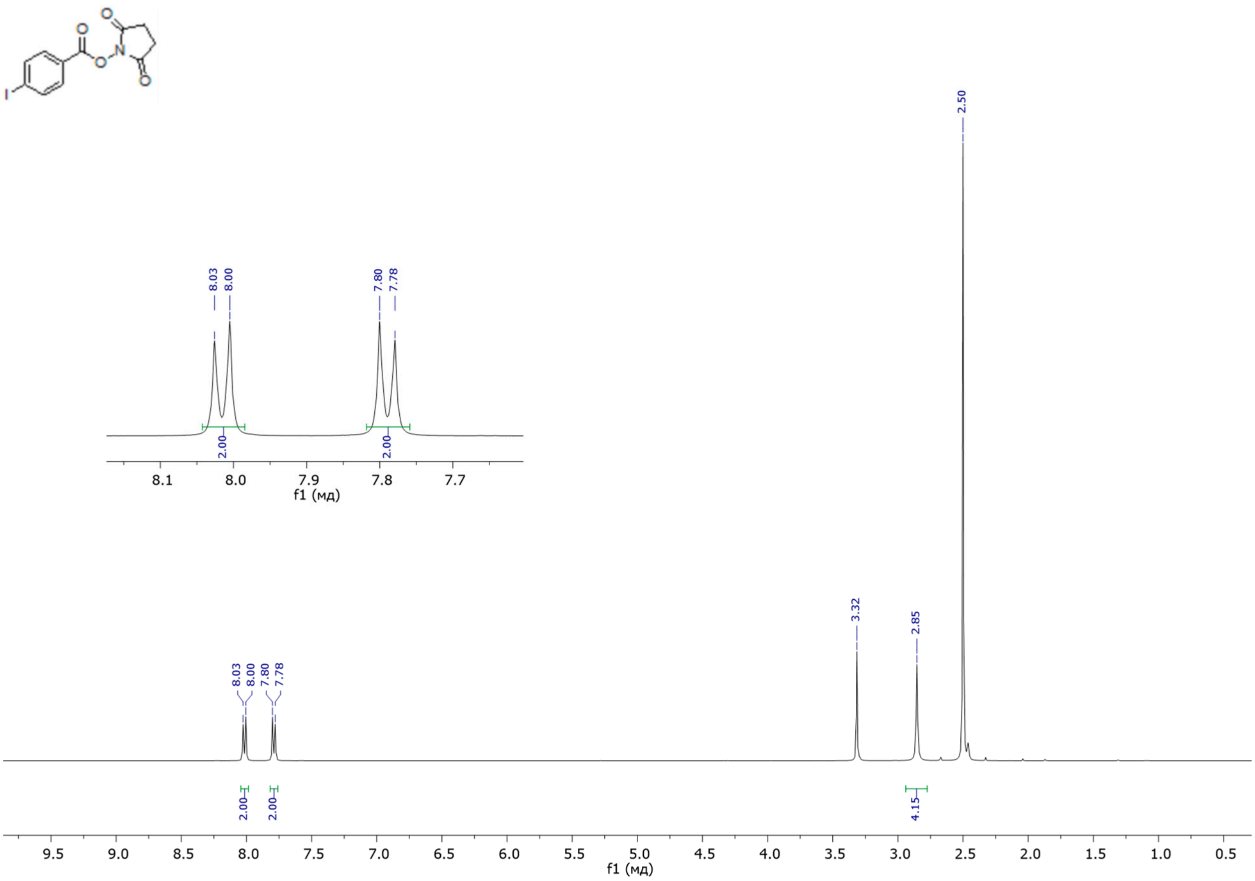

Figure S6. ^{1}H NMR spectrum for compound **2c** (400 MHz, $CDCl_3$).

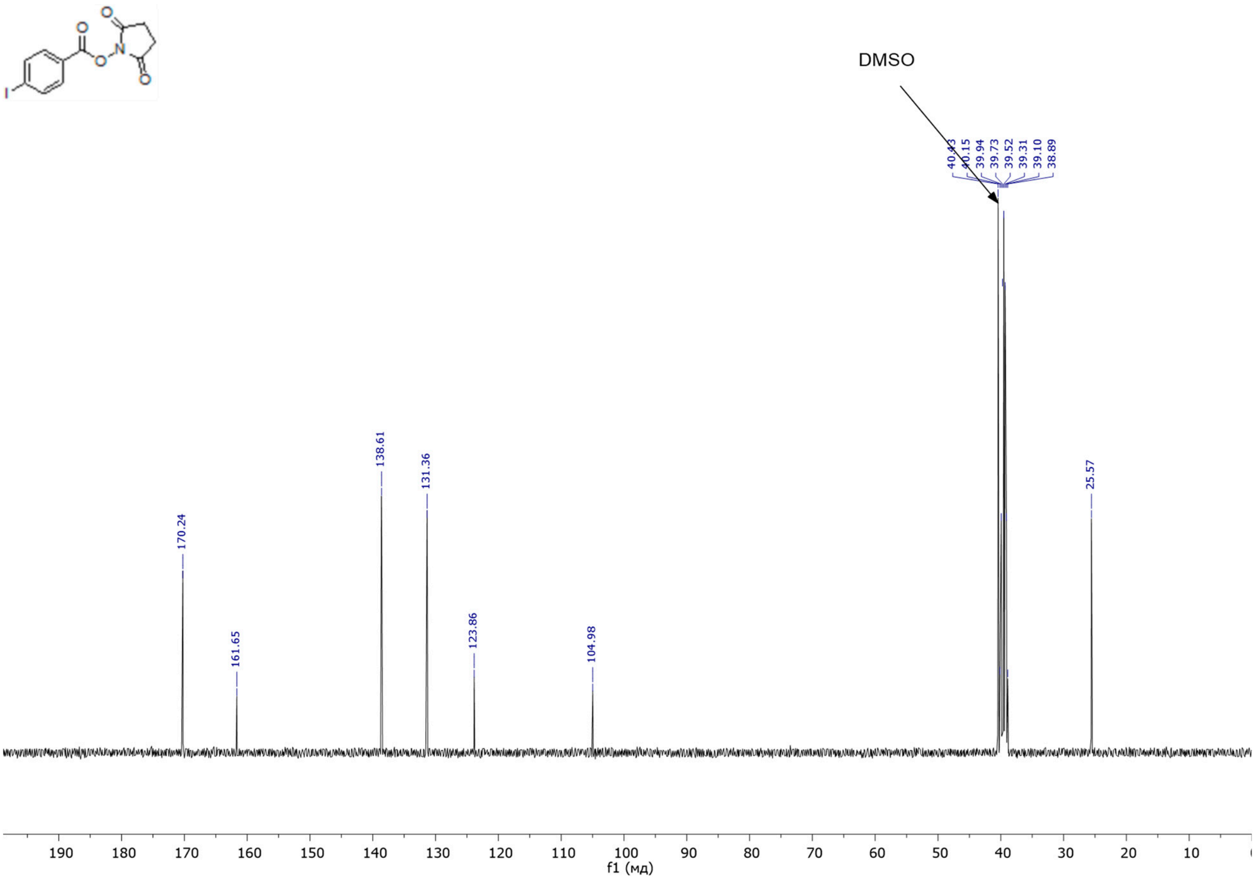

Figure S7. ^{13}C NMR spectrum for compound **2c** (101 MHz, $CDCl_3$).

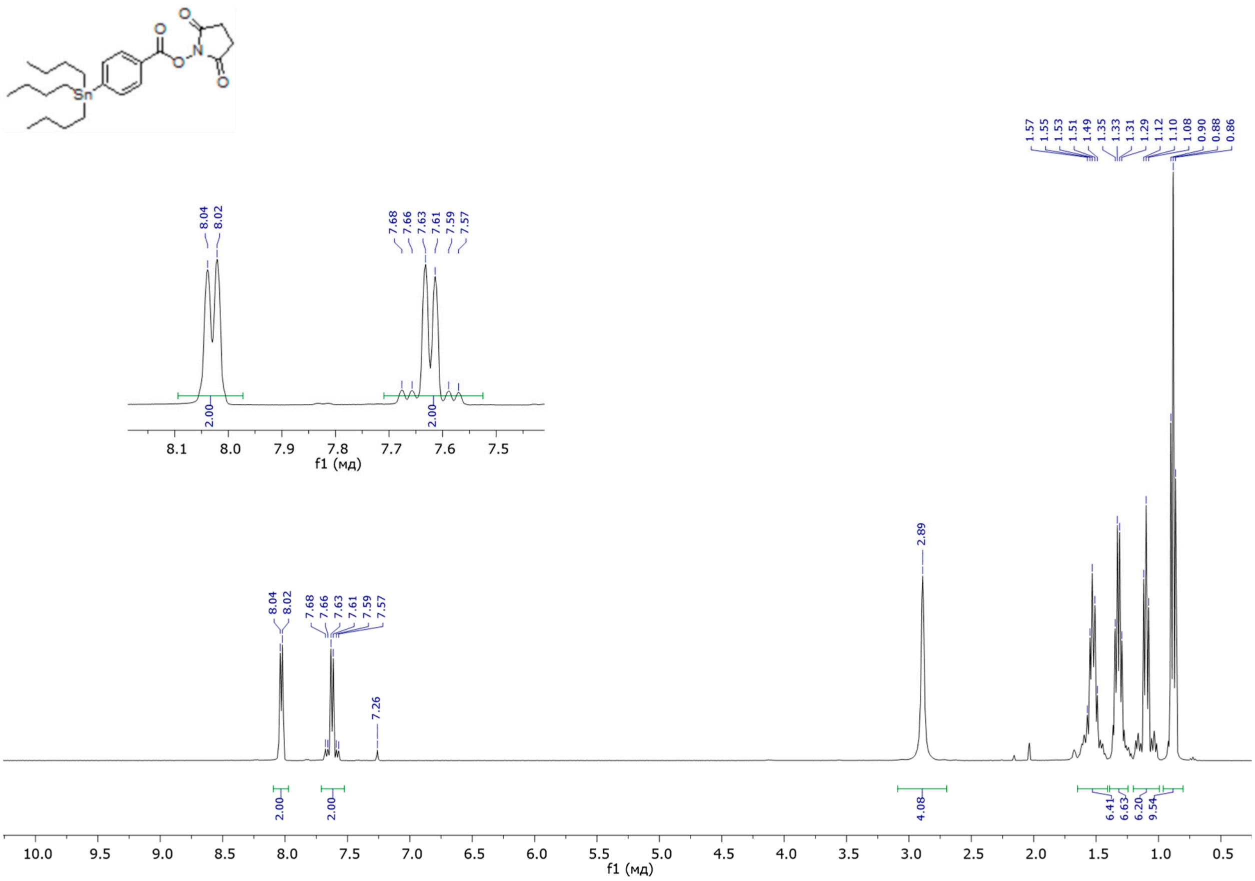

Figure S8. ^{1}H NMR spectrum for compound **3** (400 MHz, $CDCl_3$).

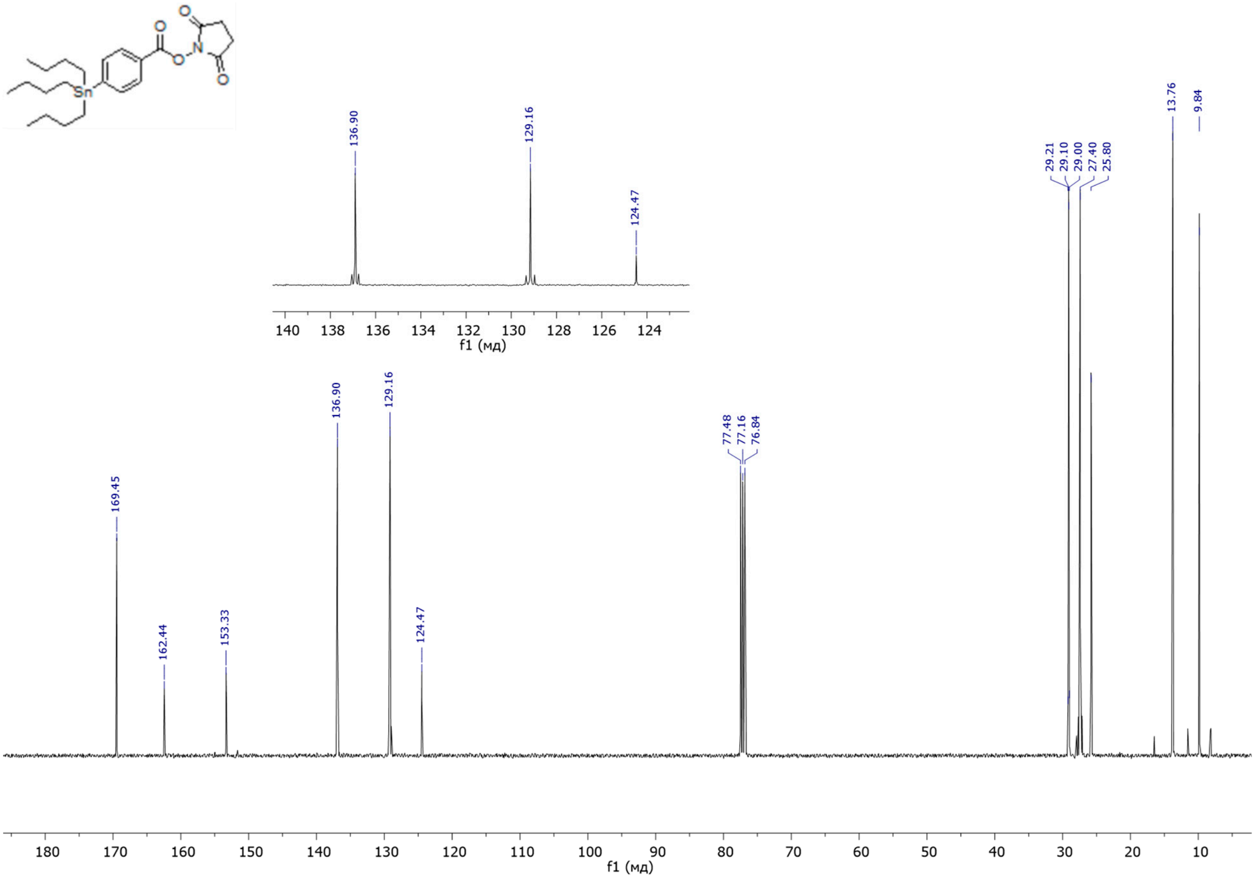

Figure S9. ^{13}C NMR spectrum for compound **3** (101 MHz, $CDCl_3$).

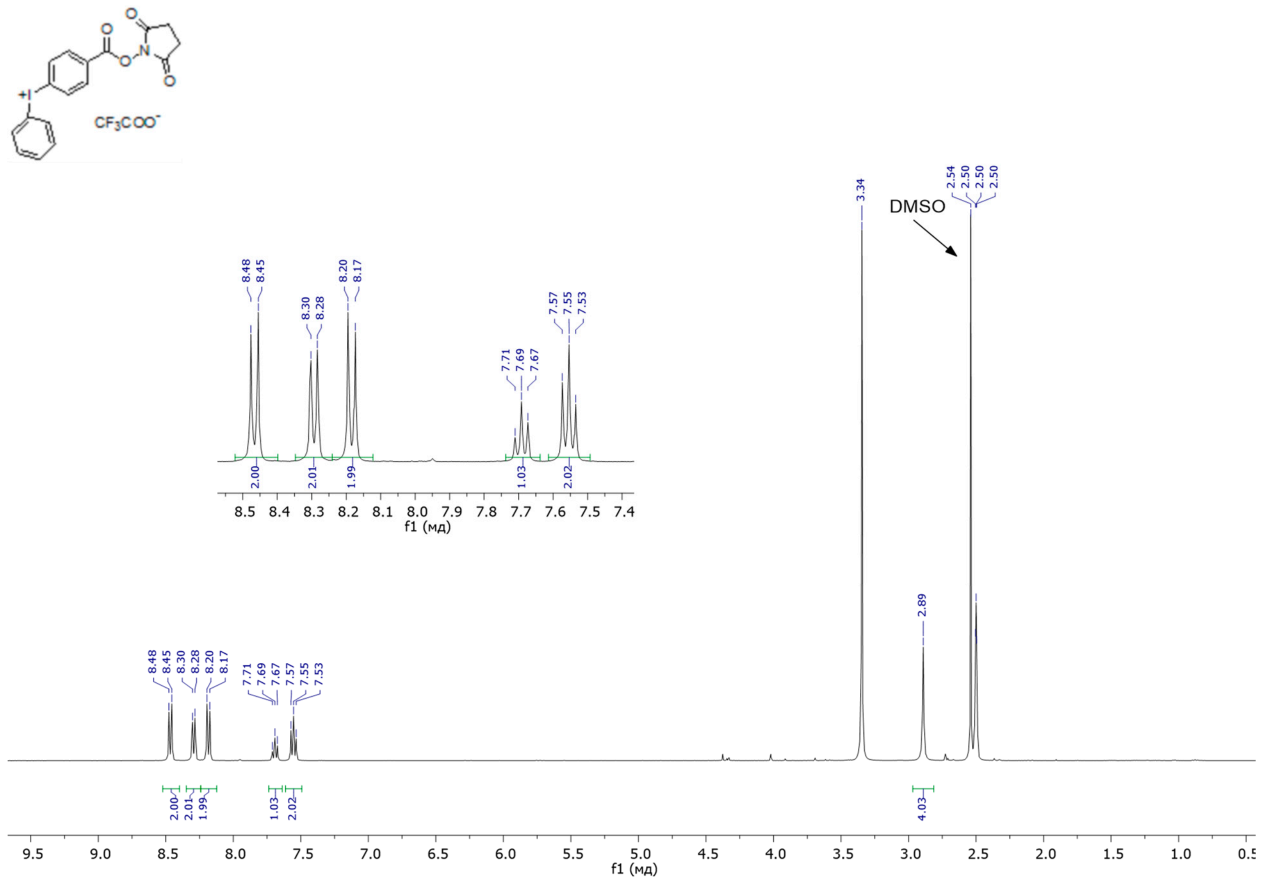

Figure S10. ^{1}H NMR spectrum for compound **4** (400 MHz, DMSO-d_6).

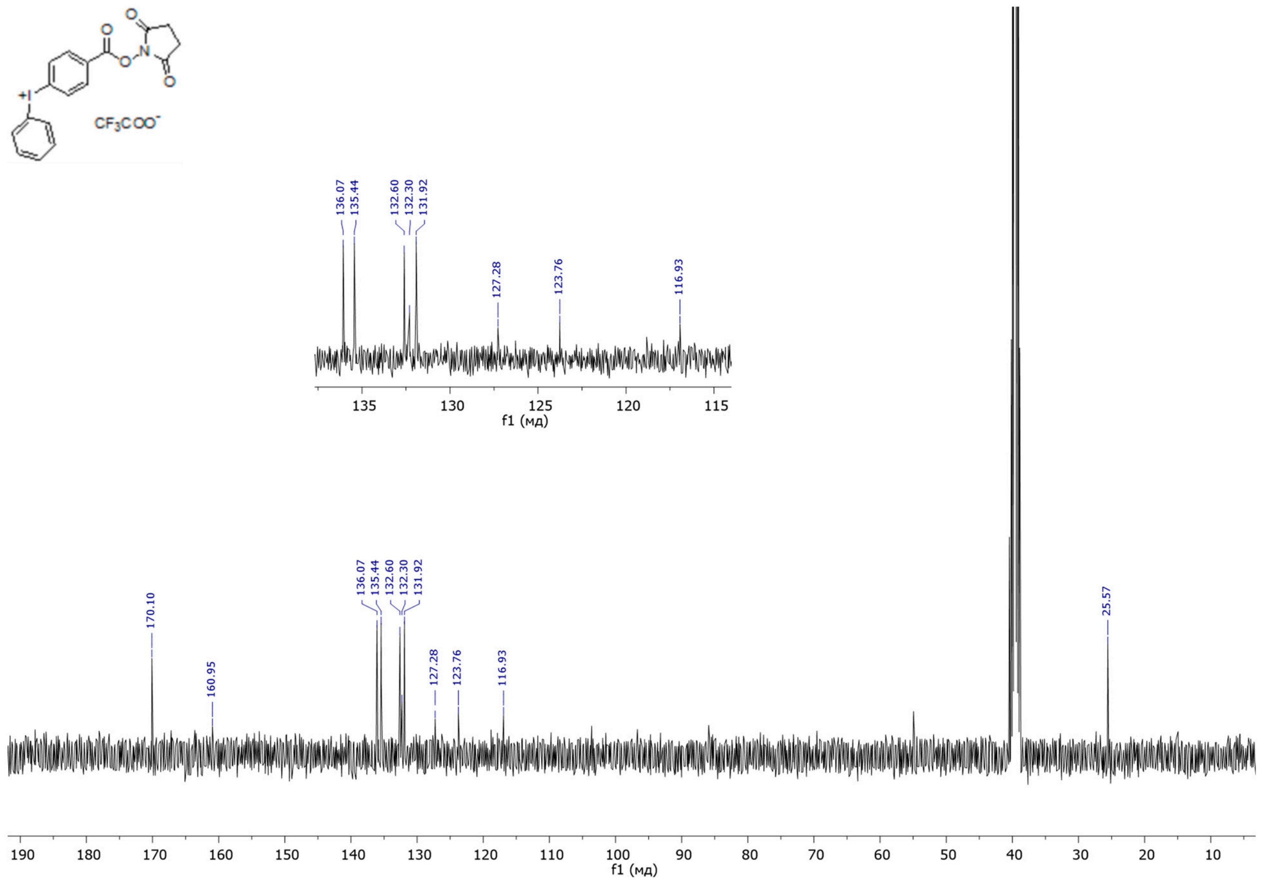

Figure S11. ^{13}C NMR spectrum for compound **4** (101 MHz, DMSO-d_6).

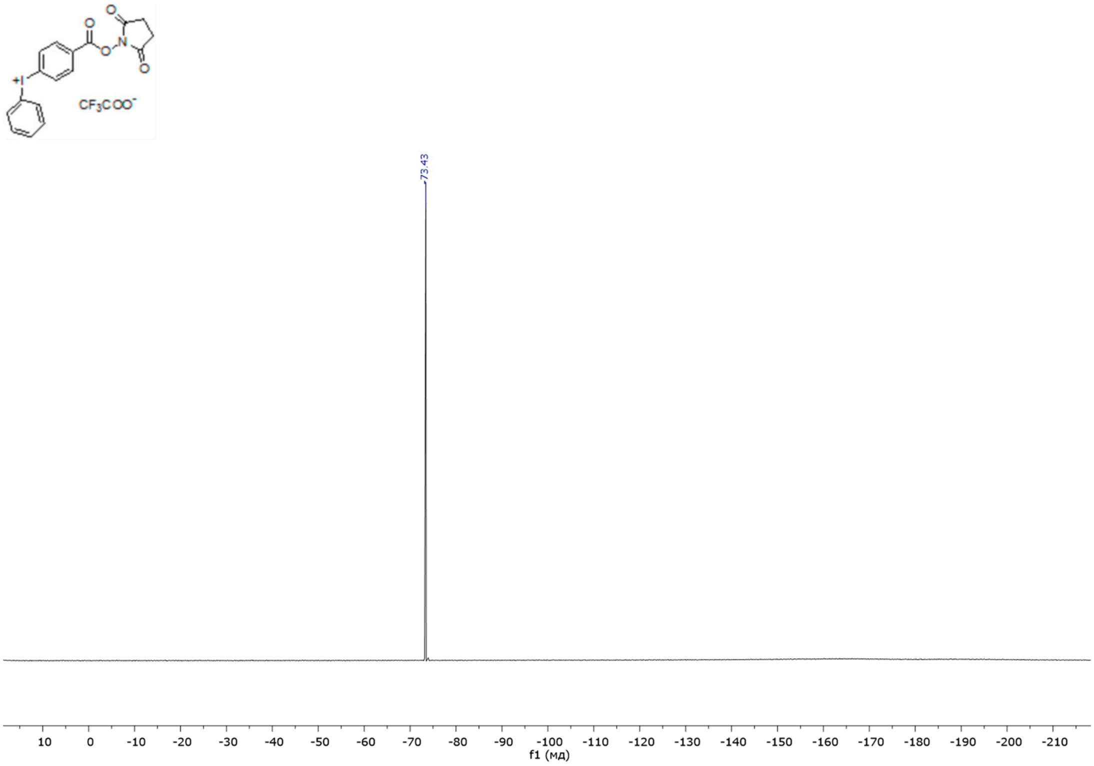

Figure S12. ^{19}F NMR spectrum for compound **4** (376.5 MHz, DMSO-d$_6$).

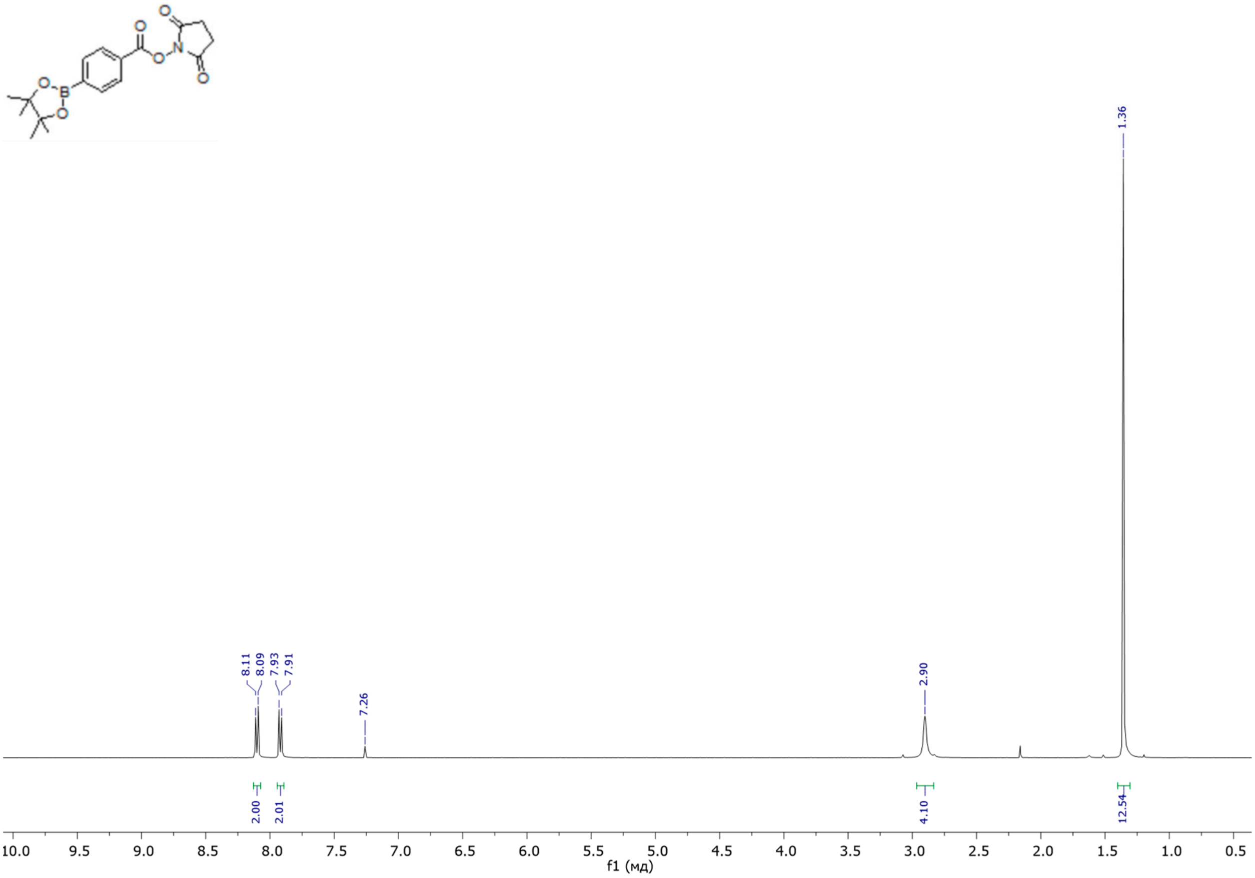

Figure S13. ^{1}H NMR spectrum for compound **6** (400 MHz, $CDCl_3$).

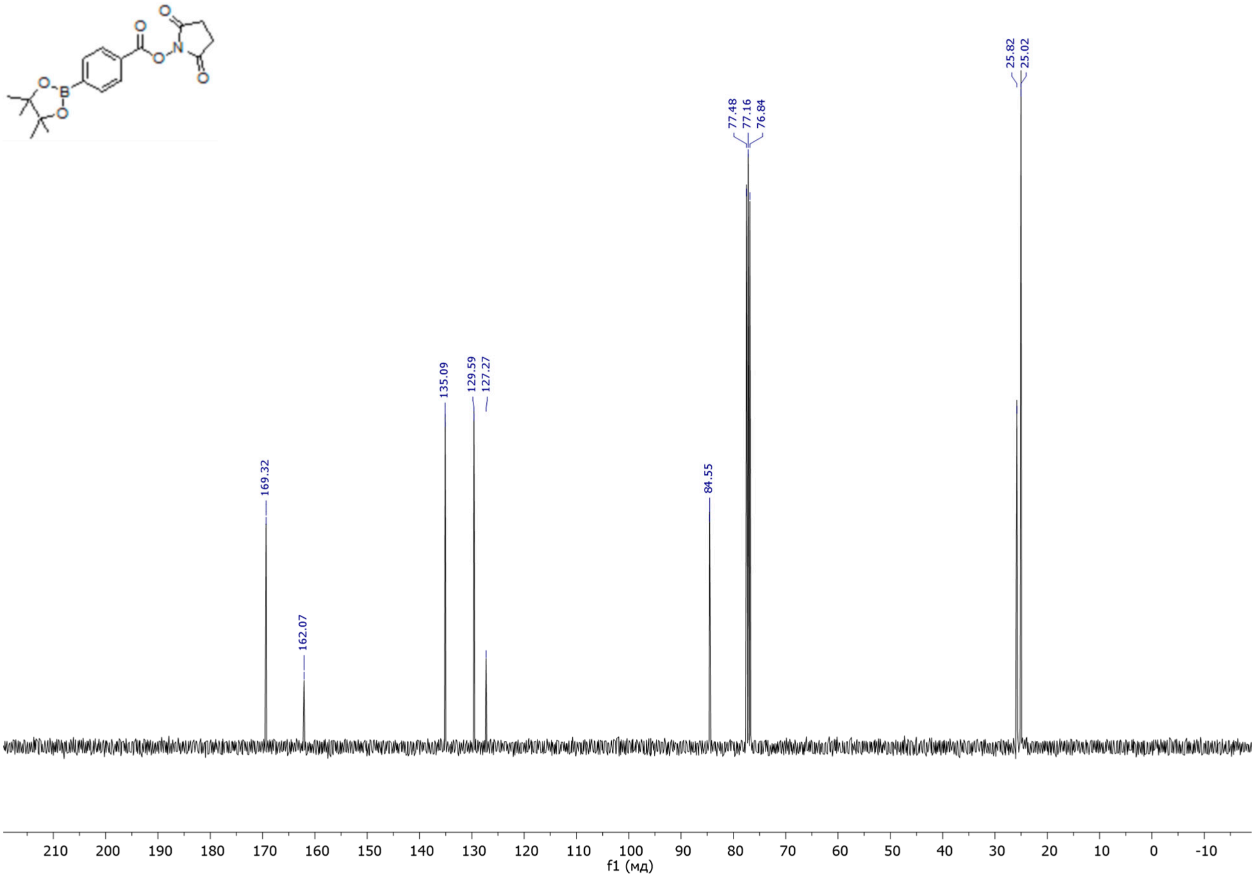

Figure S14. ^{13}C NMR spectrum for compound **6** (101 MHz, $CDCl_3$).

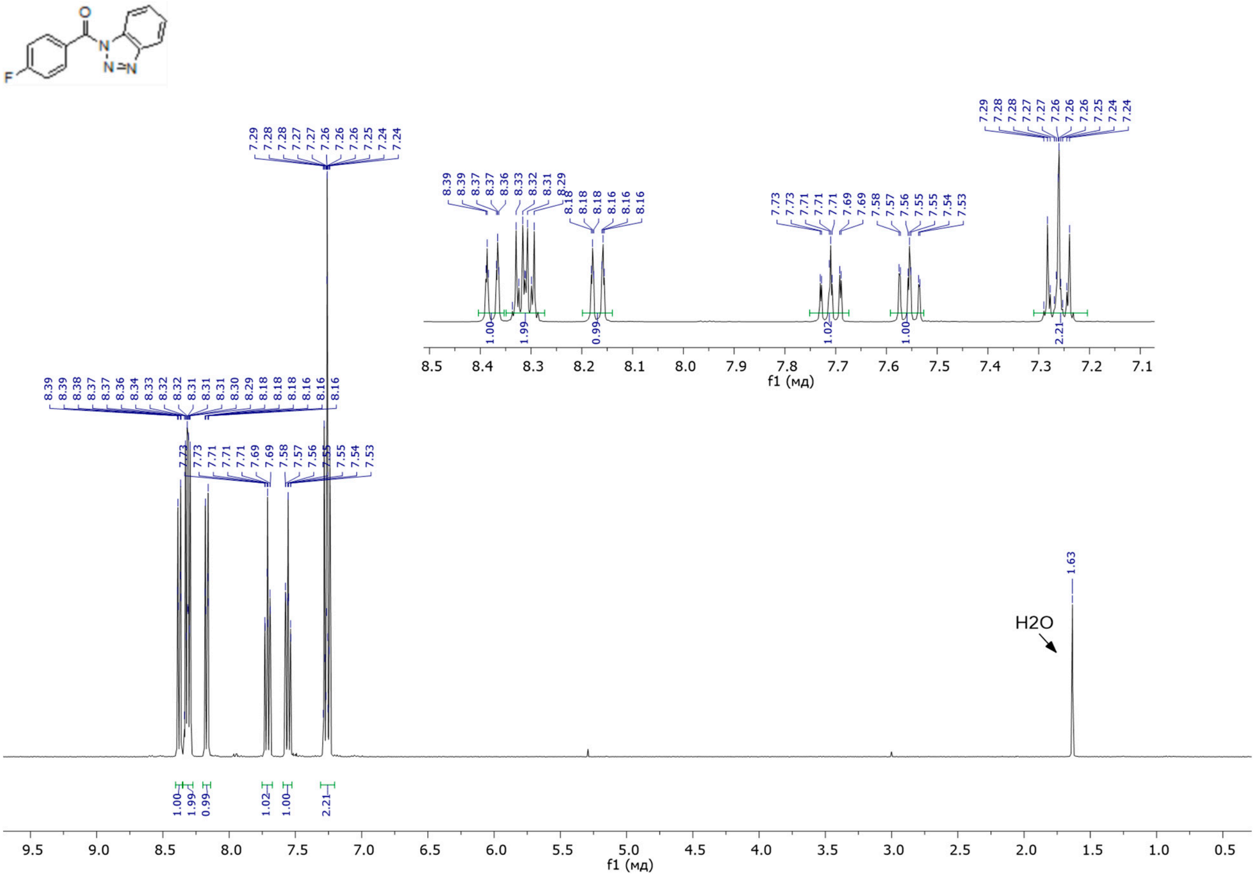

Figure S15. ^{1}H NMR spectrum for compound **7** (400 MHz, $CDCl_3$).

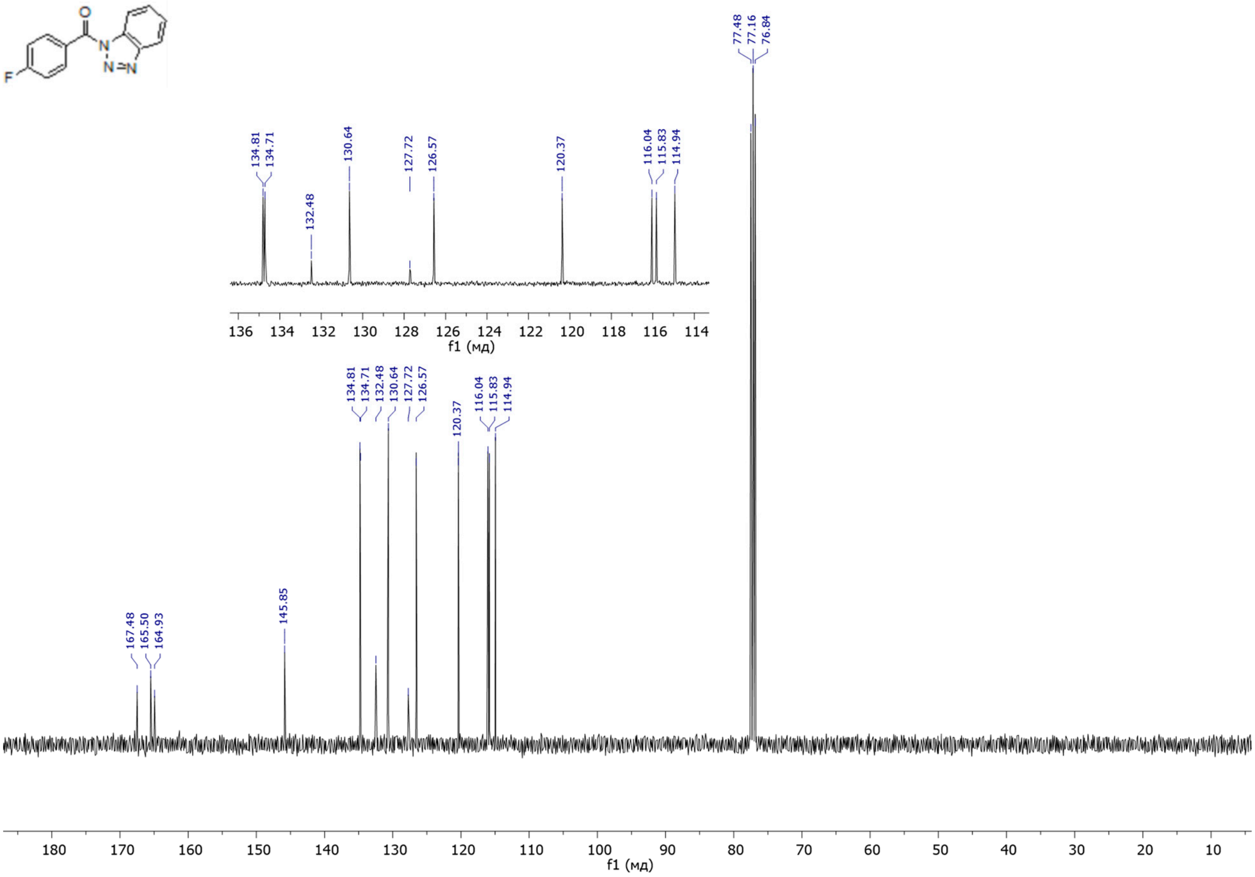

Figure S16. ^{13}C NMR spectrum for compound **7** (101 MHz, $CDCl_3$).

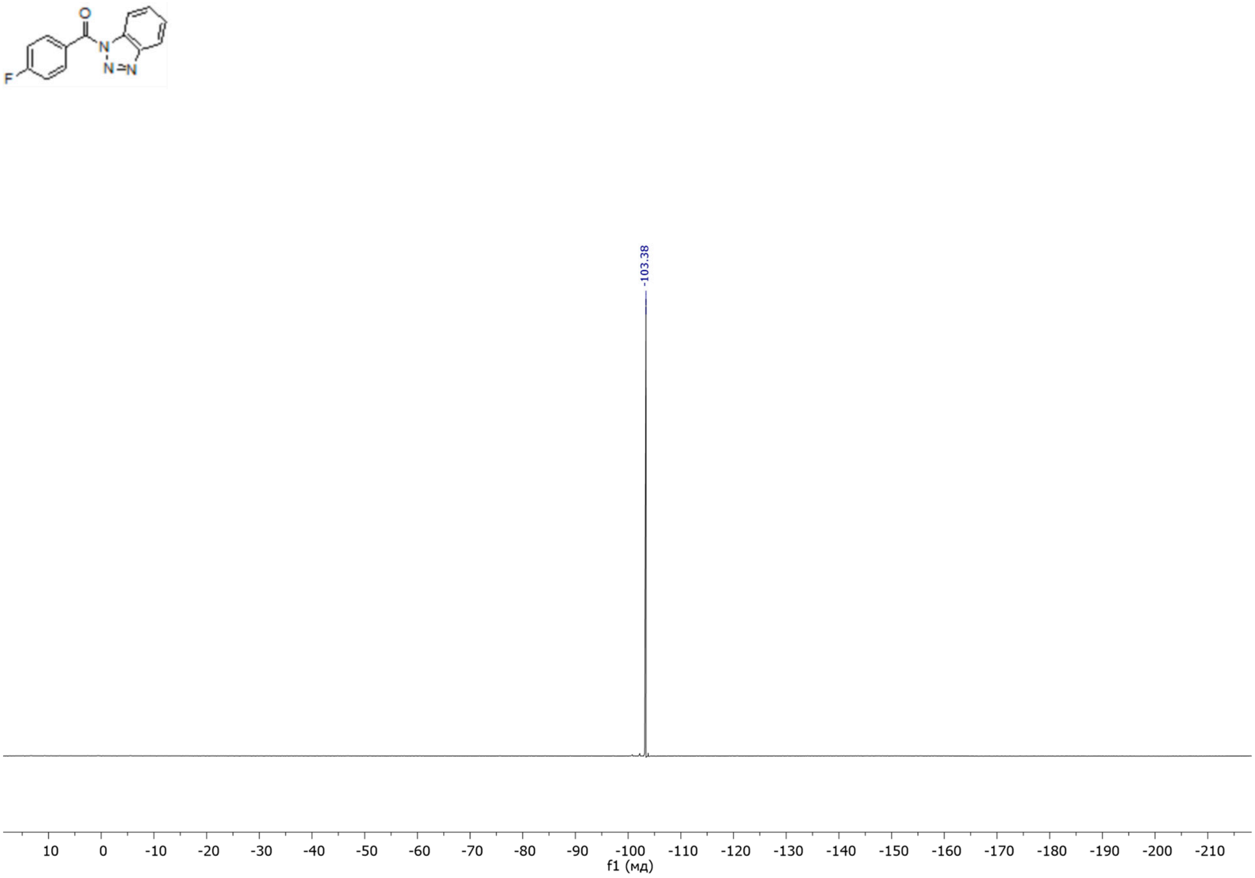

Figure S17. ^{19}F NMR spectrum for compound **7** (376.5 MHz, $CDCl_3$).

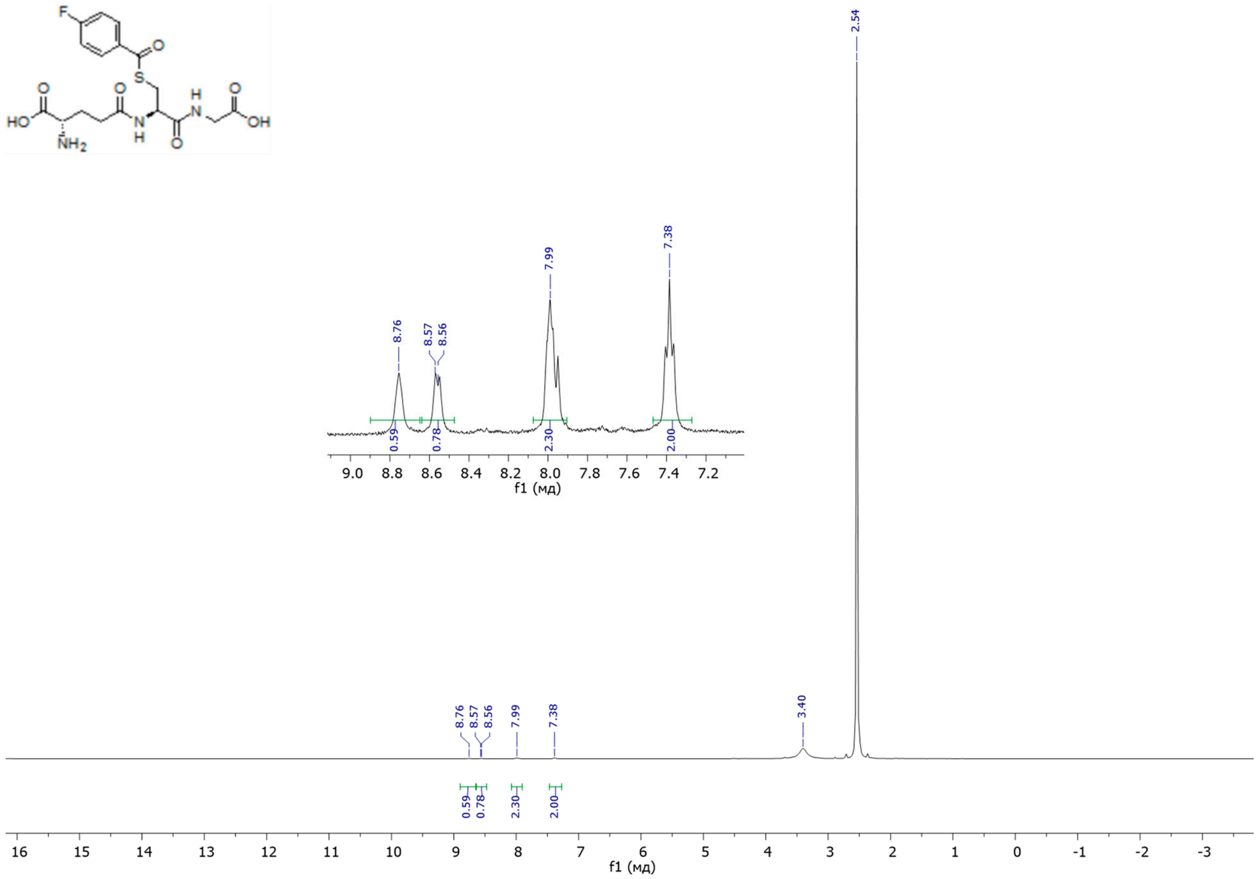

Figure S18. ^{1}H NMR spectrum for compound **([^{19}F]SFB-GSH)** (400 MHz, DMSO-d_6).

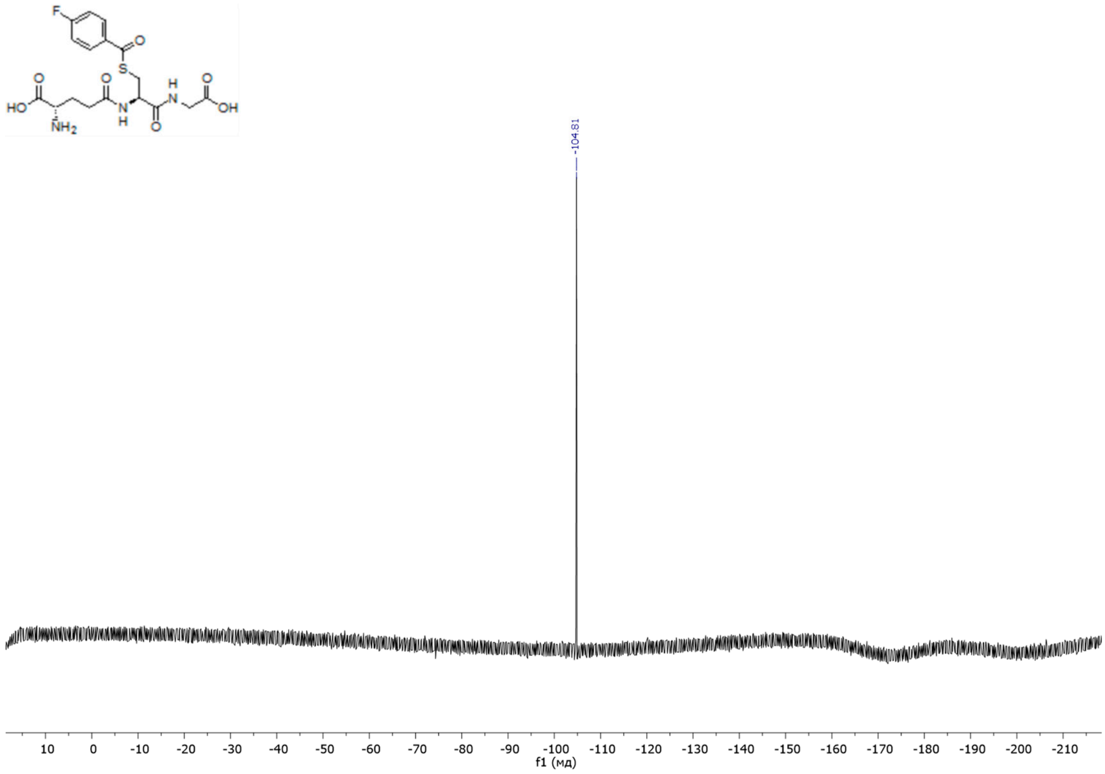

Figure S19. ^{19}F NMR spectrum for compound (**[^{19}F]SFB-GSH**) (376.5 MHz, DMSO-d_6).

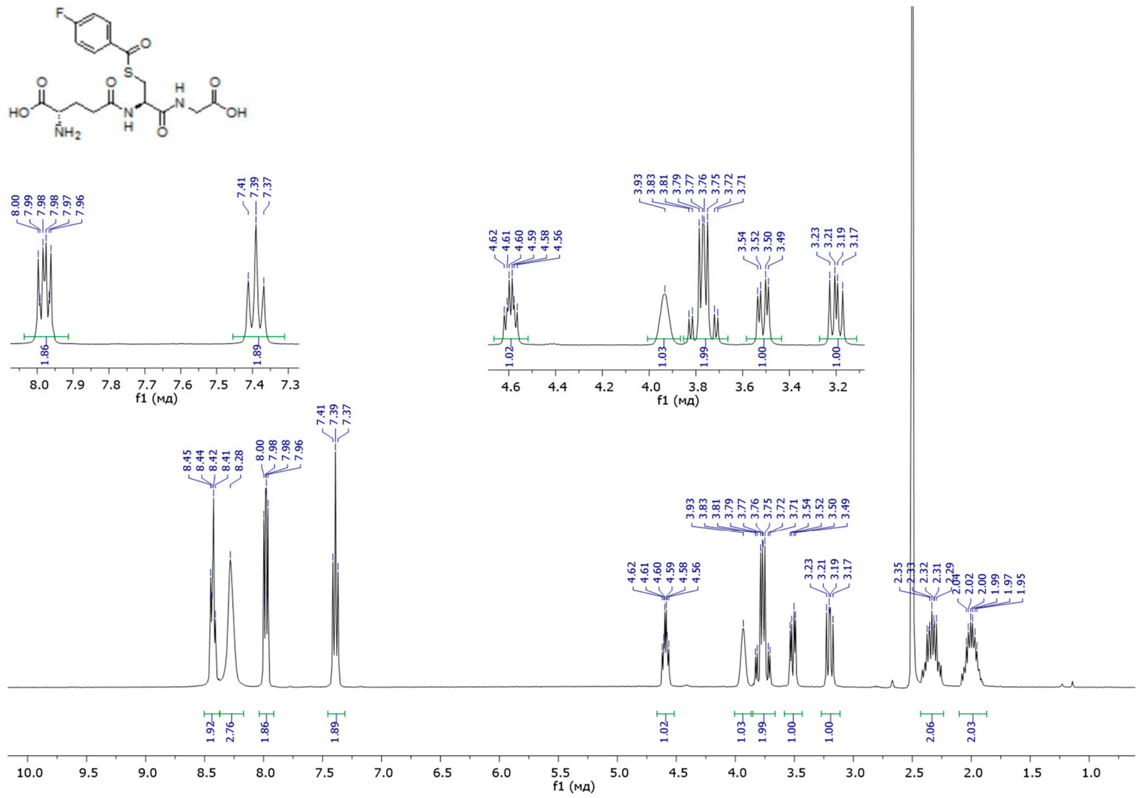

Figure S20. ^{1}H NMR spectrum for compound (**[^{19}F]SFB-GSH**) (400 MHz, DMSO-d$_6$+TFA).

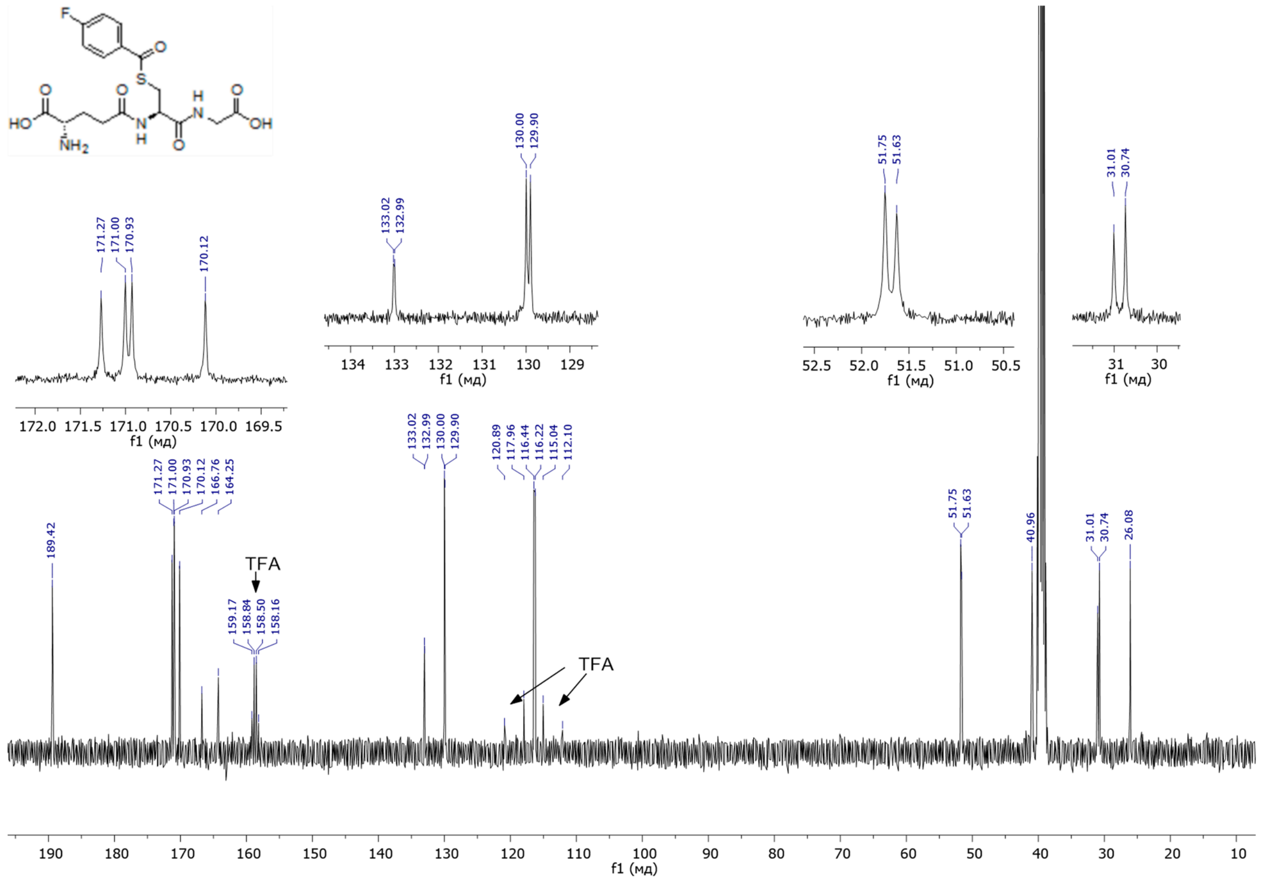

Figure S21. ^{13}C NMR spectrum for compound (**[^{19}F]SFB-GSH**) (101 MHz, DMSO-d$_6$+TFA).

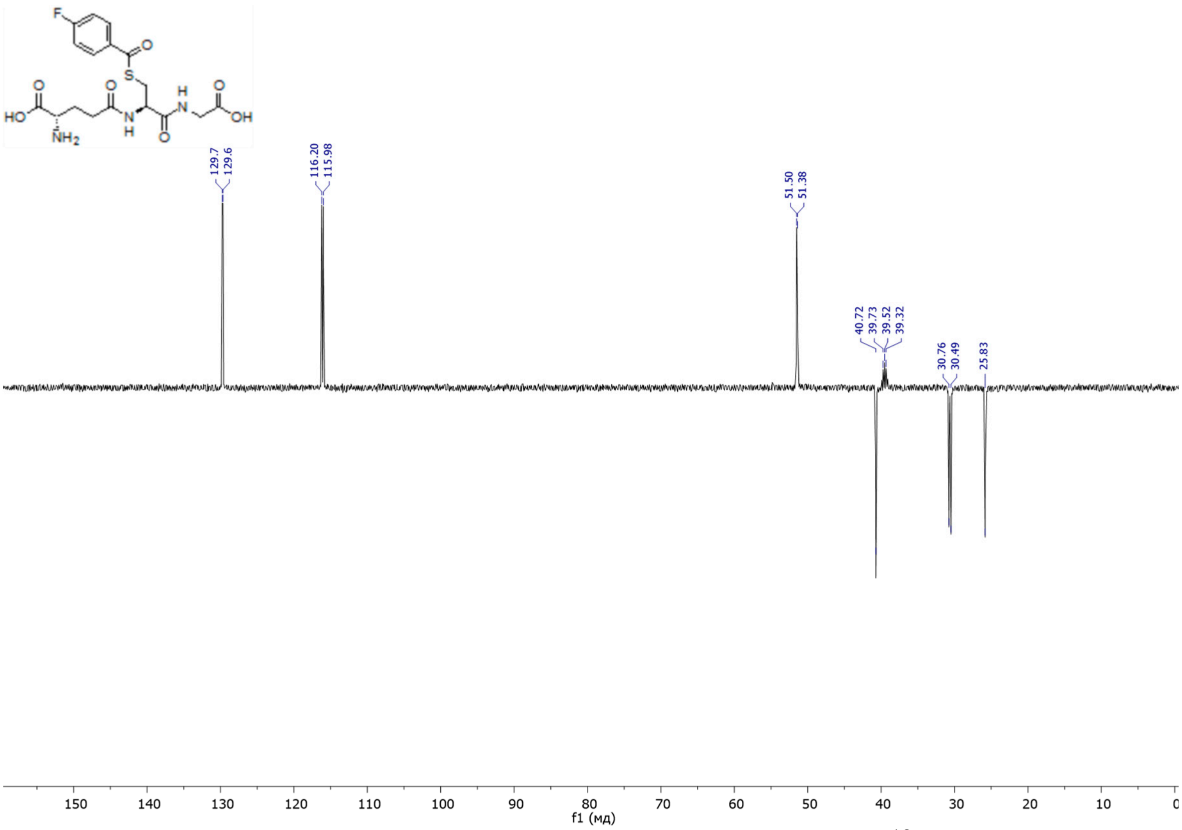

Figure S22. DEPT-135 NMR spectrum for compound (**[^{19}F]SFB-GSH)** (101 MHz, DMSO-d_6+TFA).

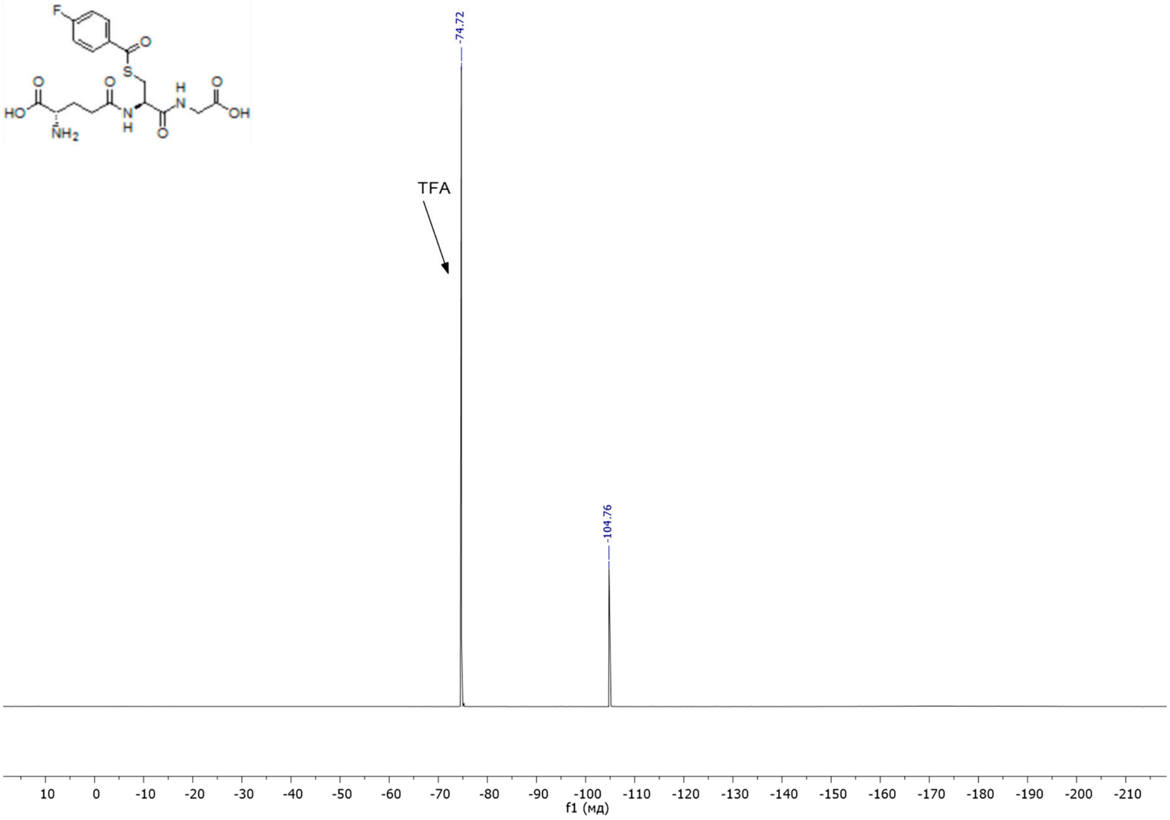

Figure S23. ^{19}F NMR spectrum for compound (**[^{19}F]SFB-GSH**) (376.5 MHz, DMSO-d_6+TFA).

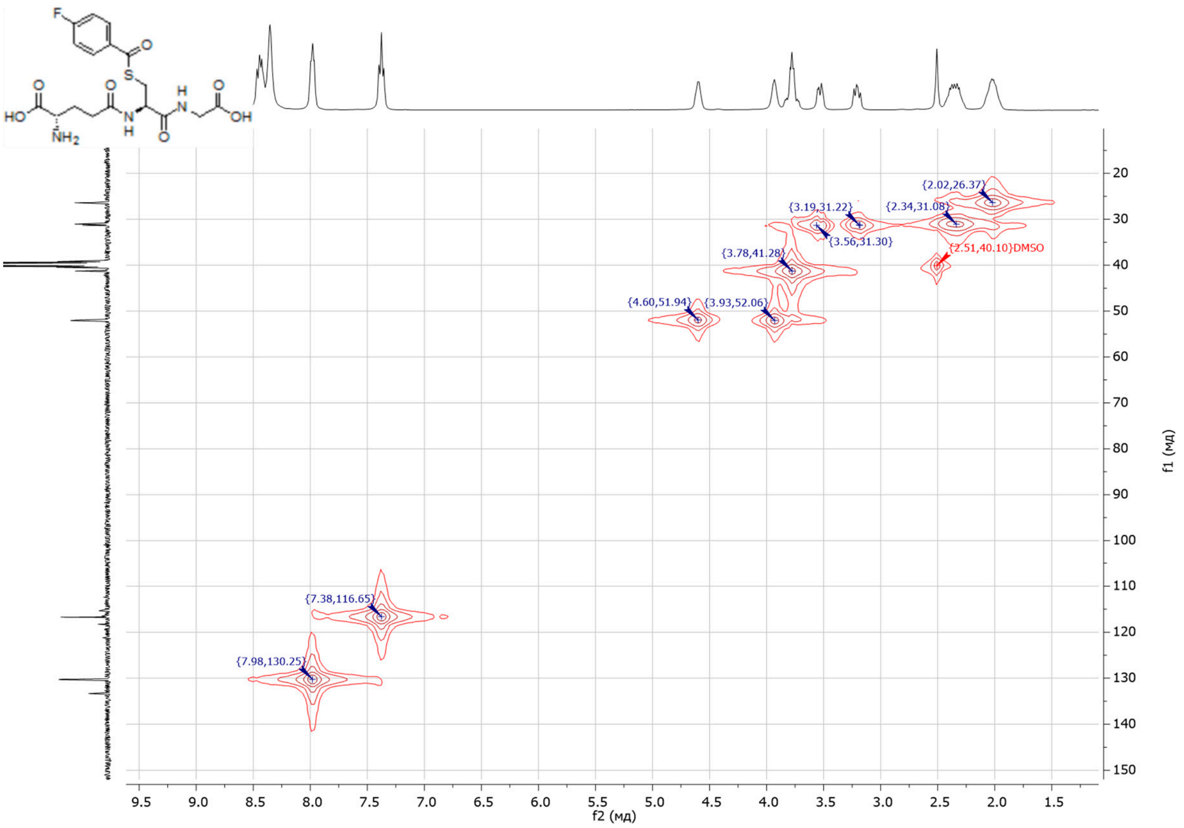

Figure S24. HSQC NMR spectrum for compound (**[19F]SFB-GSH**) (400 and 101 MHz, DMSO-d_6+TFA).

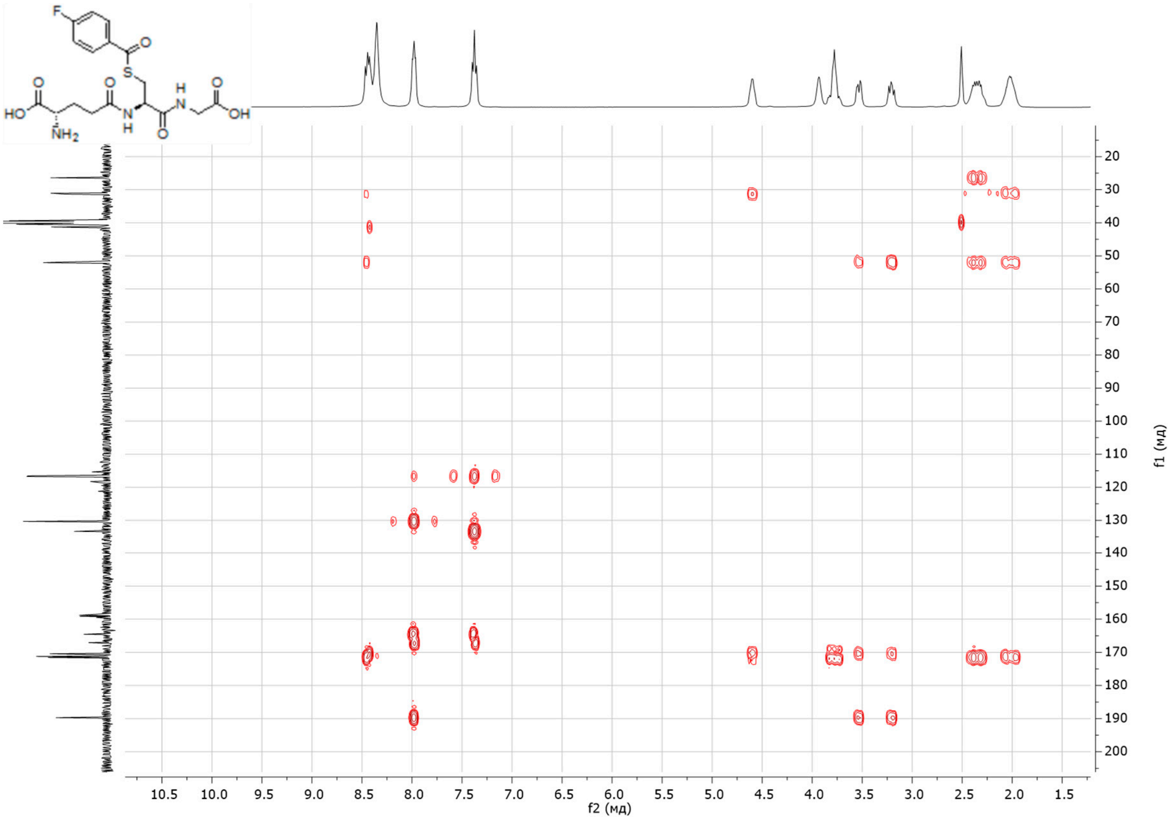

Figure S25. HMBC NMR spectrum for compound (**[19F]SFB-GSH**) (400 and 101 MHz, DMSO-d_6+TFA).

Block II. Radio chromatograms

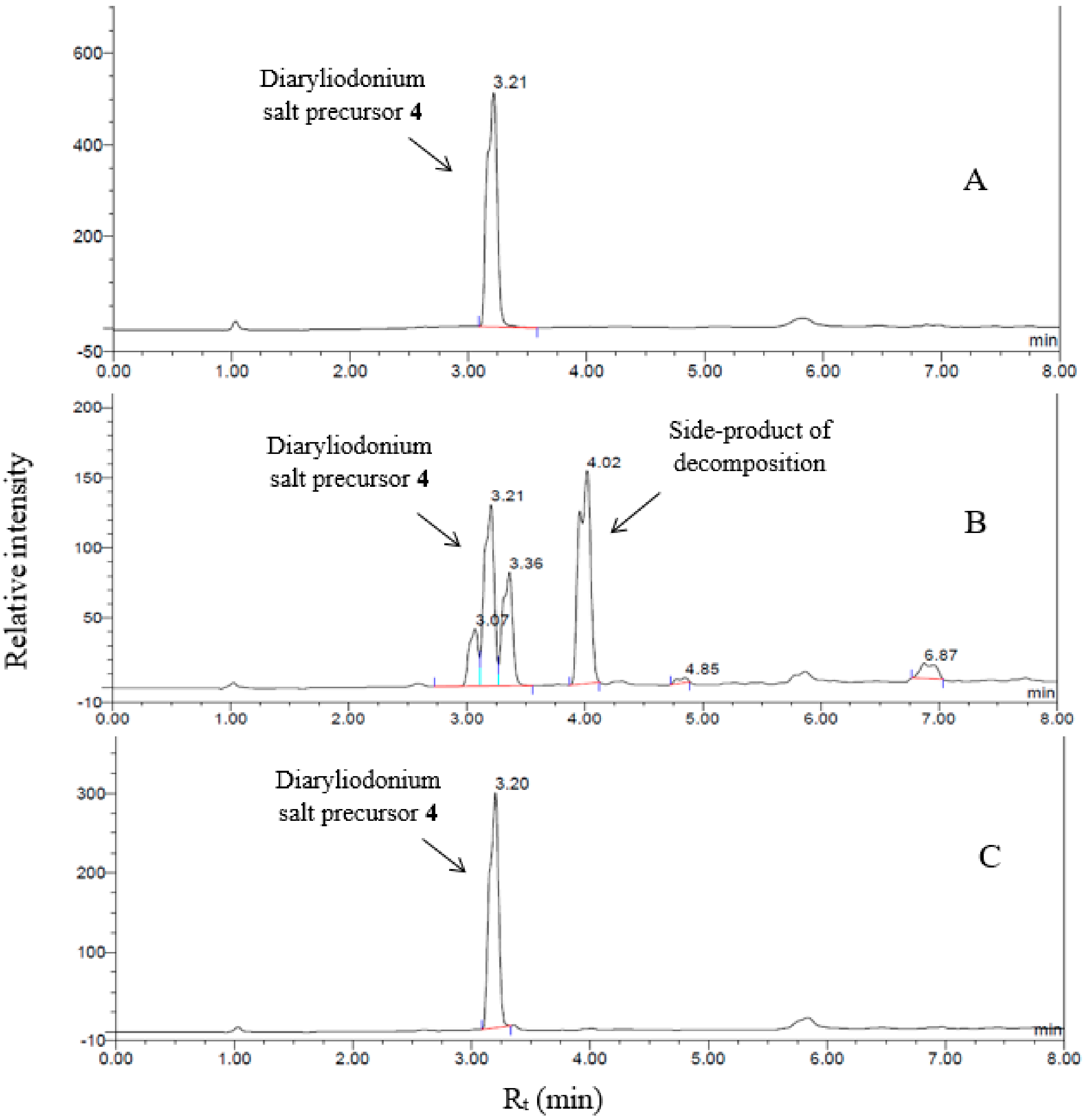

Figure S26. HPLC analysis (UV trace) of the methanolic solution of DAI precursor **4:** **A**) before elution of [^{18}F]fluoride from the cartridge; **B**) after passing through the cartridge QMA carbonate cartridge. (46 mg); **C**) after passing through Oasis WAX 1cc cartridge; HPLC column XBridge C18, 150×4.6 mm (Waters Corporation, Millford, CT, USA), eluent: 0.1% trifluoroacetic acid/acetonitrile (95/5, v/v), flow rate 2.0 mL/min, UV 254 nm.

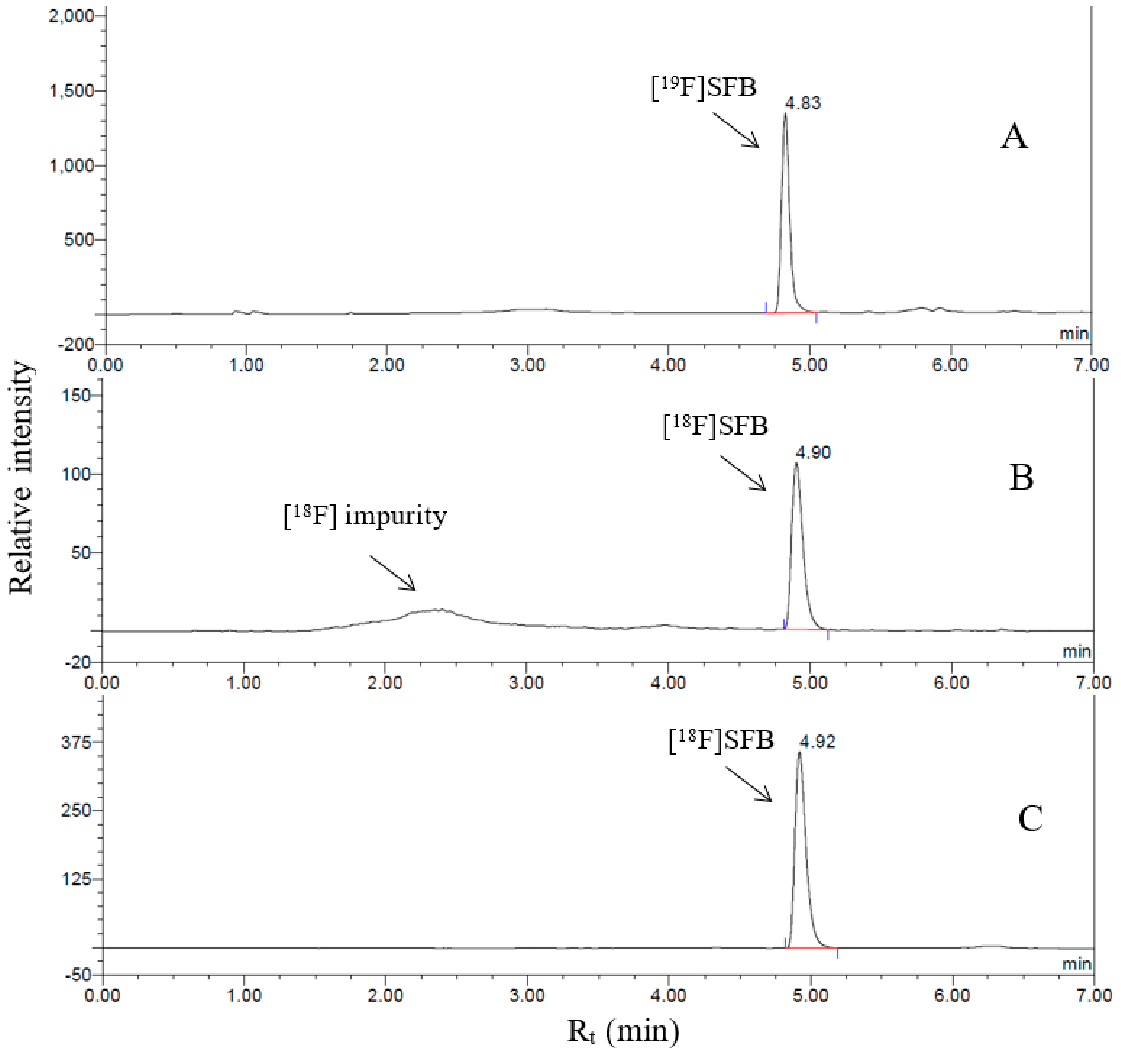

Figure S27. HPLC analysis of [^{18}F]SFB (gradient conditions, HPLC system 1, UV 254 nm). **A)** UV chromatogram of the authentic reference [^{19}F]SFB; **B)** radio-HPLC chromatogram of an aliquot of reaction mixture after radiofluorination; **C)** radio-HPLC chromatogram of [^{18}F]SFB after purification on the cartridges CM light and OASIS HLB 3cc.

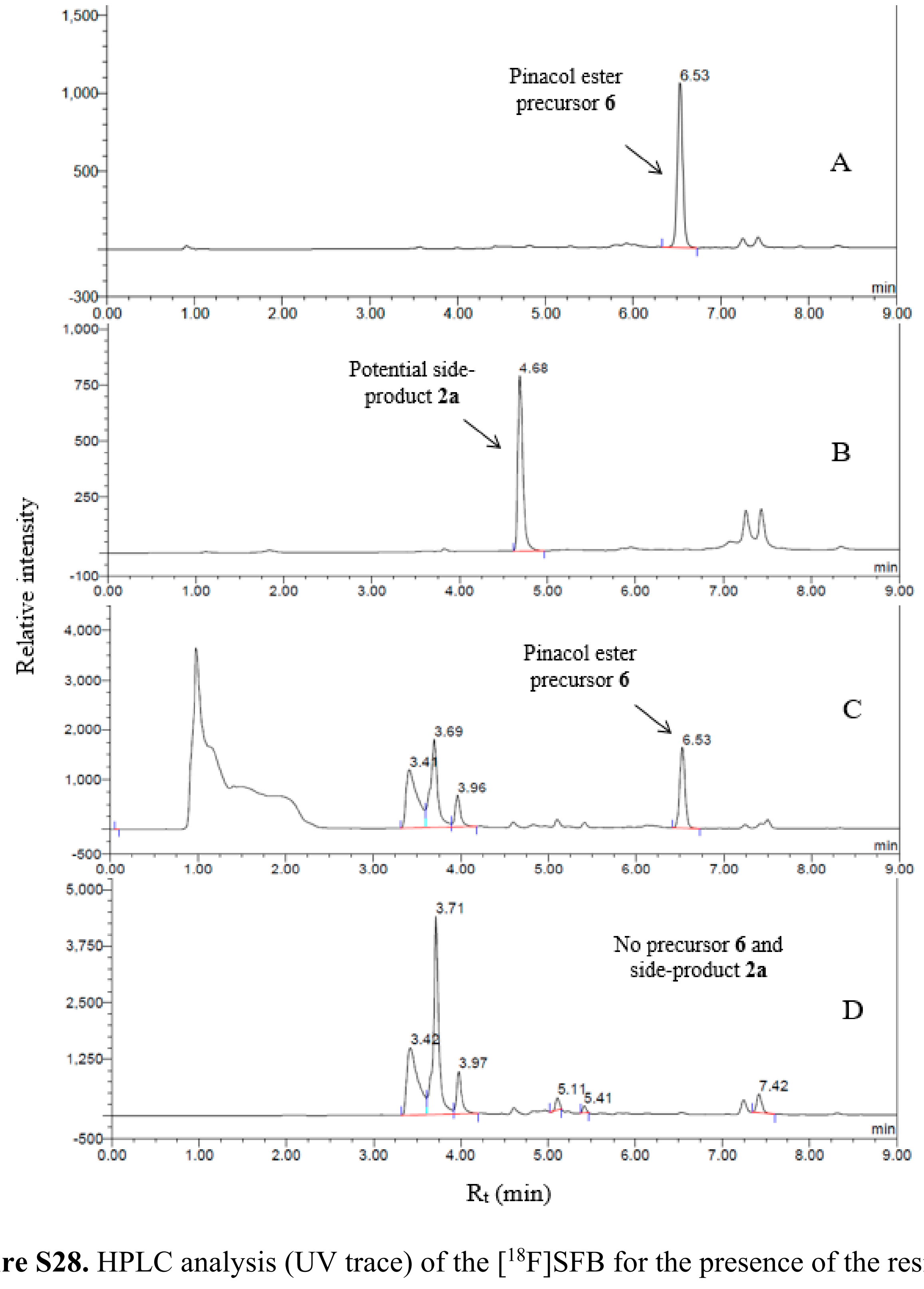

Figure S28. HPLC analysis (UV trace) of the [^{18}F]SFB for the presence of the residual amounts of **6** and potential by-product **2a**; **A)** authentic reference **6**; **B)** authentic reference **2a**; **C)** reaction mixture after radiofluorination of **6**; **D)** [^{18}F]SFB after purification on the cartridges CM light and OASIS HLB 3cc. HPLC system 1, UV 254 nm, gradient conditions.

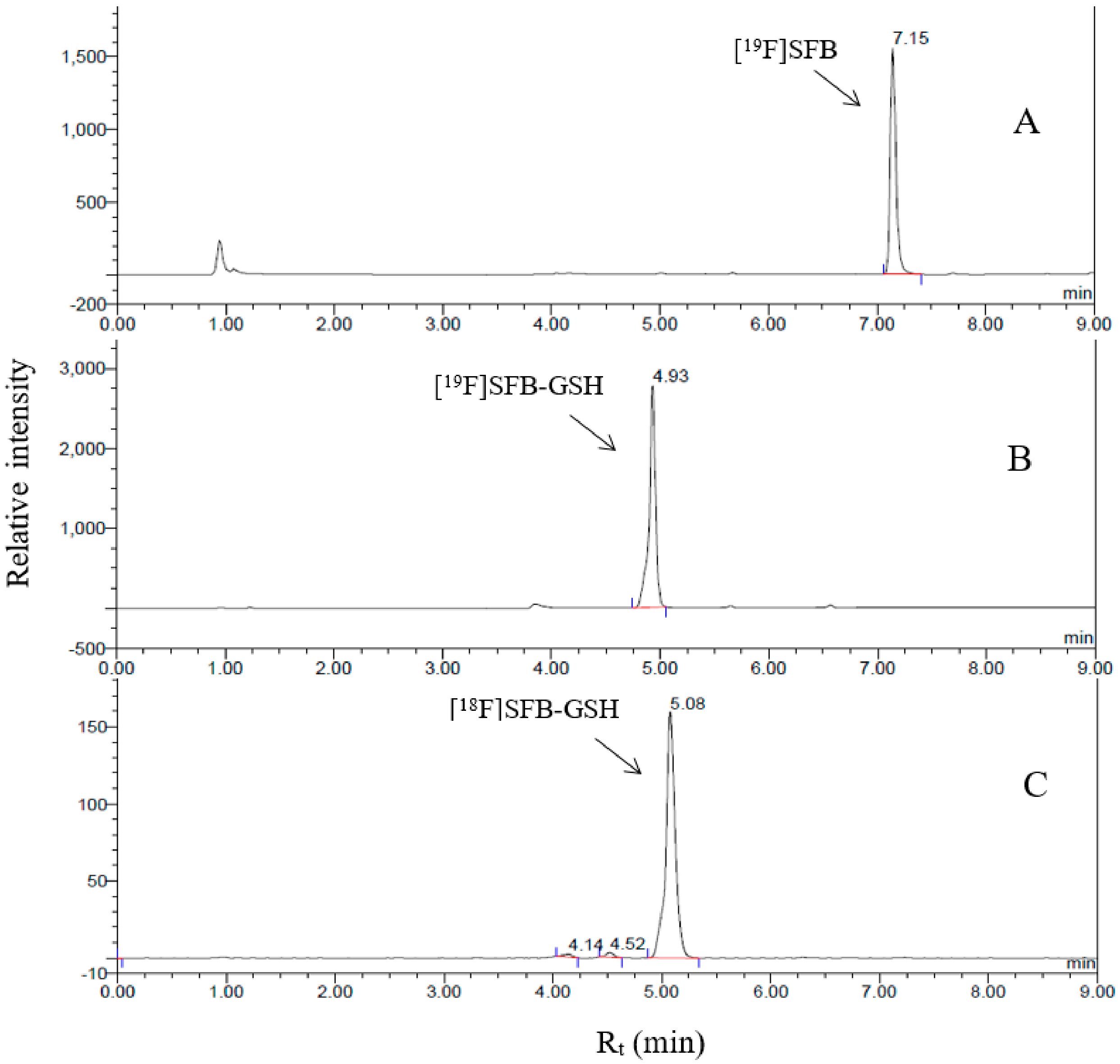

Figure S29. HPLC analysis of the [^{18}F]SFB-GSH conjugate (gradient conditions, HPLC system 2, UV 254 nm). **A)** UV trace of the authentic reference [^{19}F]SFB (**2b**); **B)** UV trace of the authentic reference [^{19}F]SFB-GSH; **C)** radio-HPLC chromatogram of the reaction mixture for [^{18}F]SFB-GSH, >99% conjugation rate.

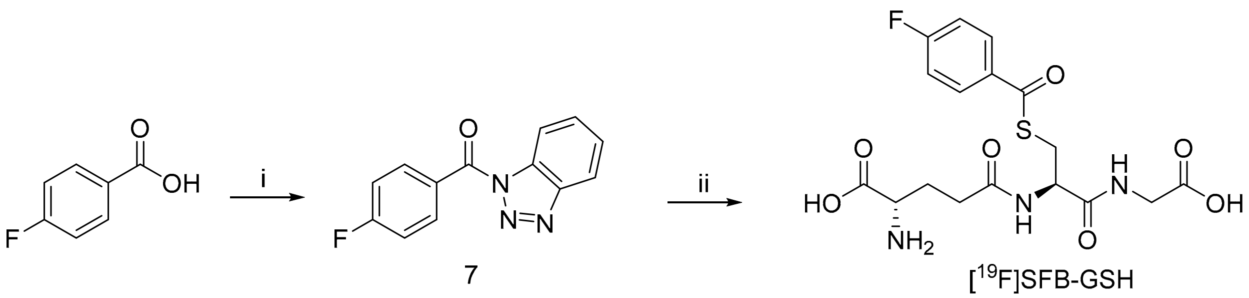

Scheme S1. Synthesis of [^{19}F]SFB-GSH. Reaction conditions. i: 1H-benzotriazole, $SOCl_2$, DCM, 20 °C, 2 h; ii: glutathione, $KHCO_3$, MeOH-H_2O, 20 °C, 20 min.
